# Adversarial Erasing Enhanced Multiple Instance Learning (siMILe): Discriminative Identification of Oligomeric Protein Structures in Single Molecule Localization Microscopy

**DOI:** 10.1002/aisy.202501159

**Published:** 2026-04-30

**Authors:** Christian Hallgrimson, Y. Lydia Li, Claire A. Shou, Ben Cardoen, John Lim, Timothy H. Wong, Ismail M. Khater, Ivan Robert Nabi, Ghassan Hamarneh

**Affiliations:** ^1^ School of Computing Science Simon Fraser University Burnaby British Columbia Canada; ^2^ Department of Cellular & Physiological Sciences, Life Sciences Institute University of British Columbia Vancouver British Columbia Canada; ^3^ Department of Electrical and Computer Engineering Faculty of Engineering and Technology Birzeit University Birzeit Palestine; ^4^ School of Biomedical Engineering University of British Columbia Vancouver British Columbia Canada

**Keywords:** caveolae, clathrin, multiple instance learning, single molecule localization microscopy, weakly supervised learning

## Abstract

Single‐molecule localization microscopy (SMLM) achieves nanoscale imaging of complex protein structures in the cell. However, the ability to capture structural variability across cell conditions (cell lines, gene expression, treatment) from 3D point cloud SMLM data remains limited. We present siMILe, a weakly supervised multiple instance learning machine learning method to close this gap in interpretable subcellular discovery. siMILe identifies condition‐specific changes in protein assemblies by leveraging their shape and network features, without requiring structure‐level supervision. siMILe improves structure classification by extending embedded instance selection through adversarial erasing and a symmetric classifier. We validated siMILe by detecting caveolae from caveolin‐1 (Cav1) labeled PC3 prostate cancer cells differentially expressing cavin‐1. In PC3‐CAVIN1 cells, cavin‐1 closely associates with siMILe‐identified caveolae, to a lesser extent with higher‐order noncaveolar Cav1 scaffolds, but not small Cav1 oligomers corresponding to 8S complexes, supporting a role for progressive cavin‐1 interaction in 8S complex oligomerization. We also validated siMILe on simulated SMLM data and in detecting inhibitor‐induced structural variations within clathrin‐coated pit data. These results highlight siMILe's potential to identify differential molecular structures in distinct cell conditions. siMILe extends the SuperResNET SMLM software platform with the ability to detect interpretable structural differences across conditions.

## Graphical abstract

Hallgrimson et al. introduce a machine learning algorithm, siMILe, that takes features of single‐molecule localization microscopy localization clusters (e.g., size and sphericity) and finds the clusters that are associated with certain cell conditions (such as differential protein expression or drug treatment). siMILe was used to find discriminatory clusters of caveolin‐1 and clathrin, two proteins that form invaginations at the plasma membrane.

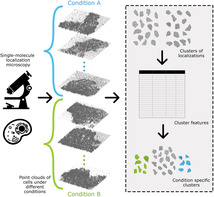

## 1 Introduction

Single‐molecule localization microscopy (SMLM) is a super‐resolution microscopy technique that achieves superior resolution based on detection of the stochastic blinking of isolated fluorophores [1]. SMLM achieves 20 nm lateral resolution, compared to the ∼250 nm resolution limit encountered by conventional microscopy [2], with more recent SMLM‐based approaches such as MinFlux achieving 1–3 nm 3D resolution [1, 3]. The application of machine learning algorithms to 3D SMLM has increased the efficacy and accuracy of the reconstruction of super‐resolution images from point emitter frames and for background fluorescence classification [4, 5]. However, approaches for quantitative analysis of 2D or 3D point‐cloud data remain limited. Cluster analysis methods, including statistical, Bayesian, density‐based, correlation‐, tessellation‐, image‐, and machine‐learning based approaches, have been applied to SMLM data [6, 7, 8]. The SuperResNET network analysis software platform leverages the power of graph‐based construction and advanced machine learning techniques to process and interpret the complex point cloud data generated by SMLM [9]. Previously, we applied SuperResNET to caveolin‐1 (Cav1) point clouds to classify caveolae and smaller noncaveolar oligomeric structures, called scaffolds [10] and detected caveolae and three classes of scaffolds as well as structural changes induced by point mutations to the Cav1 scaffolding domain [9, 11, 12]. More recently, SuperResNET was able to detect changes to clathrin‐coated pits induced by small molecule inhibitors of clathrin endocytosis, which were not observed by transmission electron microscopy, and effectively interpreted MinFlux clathrin pits and vesicles [13]. SuperResNET analysis of publicly available 2D SMLM data for nucleoporin Nup96 effectively segmented nuclear pores and Nup96 corners and distinguished 2 modules within the corners at 10.7±0.1 nm distance, thereby achieving molecular resolution [14]. Therefore, SuperResNET represents a highly sensitive approach to studying the spatial or molecular architecture of subcellular structures in situ in the intact cell.

The primary strengths of SuperResNET lie in its ability to analyze the network structure inherent in SMLM data and its integration of advanced machine learning algorithms for pattern recognition and structure identification. These features enable SuperResNET to uncover subtle structural patterns and relationships that might be overlooked by conventional analysis methods. The use of prior knowledge, in the form of defined group labels, enabled identification by SuperResNET of diverse Cav1 structures showing that the base Cav1 oligomer (the so‐called 8S complex) combines to form higher‐order scaffolds and caveolae [9, 15]. PC3 prostate cancer cells that express Cav1, but not the adaptor protein cavin‐1 (also called PTRF) required for caveolae formation, were compared with PC3‐CAVIN1 cells transfected with cavin‐1 that now present caveolae, defining parameters that enabled identification of known structures, the 8S complex and caveolae, and thereby previously unknown intermediate higher order oligomers [9]. SuperResNET has also been used to study the spatial relationship on caveolae of the adaptor proteins EHD2 and PASCIN2 [16].

Here we build on SuperResNET to present a discovery method named siMILe, a weakly‐supervised, machine learning algorithm designed to identify discriminatory changes in mesoscale domain structures between conditions. The idea is to leverage only weak labels: the image‐ or cell‐level information, such as cell type, epigenetic, or environmental interventions [17]. “Strong” labels in this context would be object‐ or structure‐level annotations, which in SMLM often are absent.

Such a problem statement is frequently tackled by multiple instance learning (MIL) methods. Unlike traditional supervised learning techniques, where labels are assigned to each object, or “instance”, with MIL, the assumption is that only a set, or collection of instances, called “bags”, can be given a label. This is particularly effective when individual instances cannot be labeled, whether infeasible or expensive. It is important to note that this differs from quantifying the structural diversity or heterogeneity [18, 19], given that discovery of diversity does not in itself leverage weak labels to find discriminative differences. MIL has been applied to many fields, ranging from confocal microscopy [20], drug efficacy discovery [21], DNA protein identification, and histopathology classification [13]. A more recent proposed MIL approach [22] optimizes its output so that nearby objects have the same or a similar object label. While this is a valid constraint in several MIL applications, here it would not be appropriate because in SMLM proximate protein complexes can be quite dissimilar.

**FIGURE 1:**
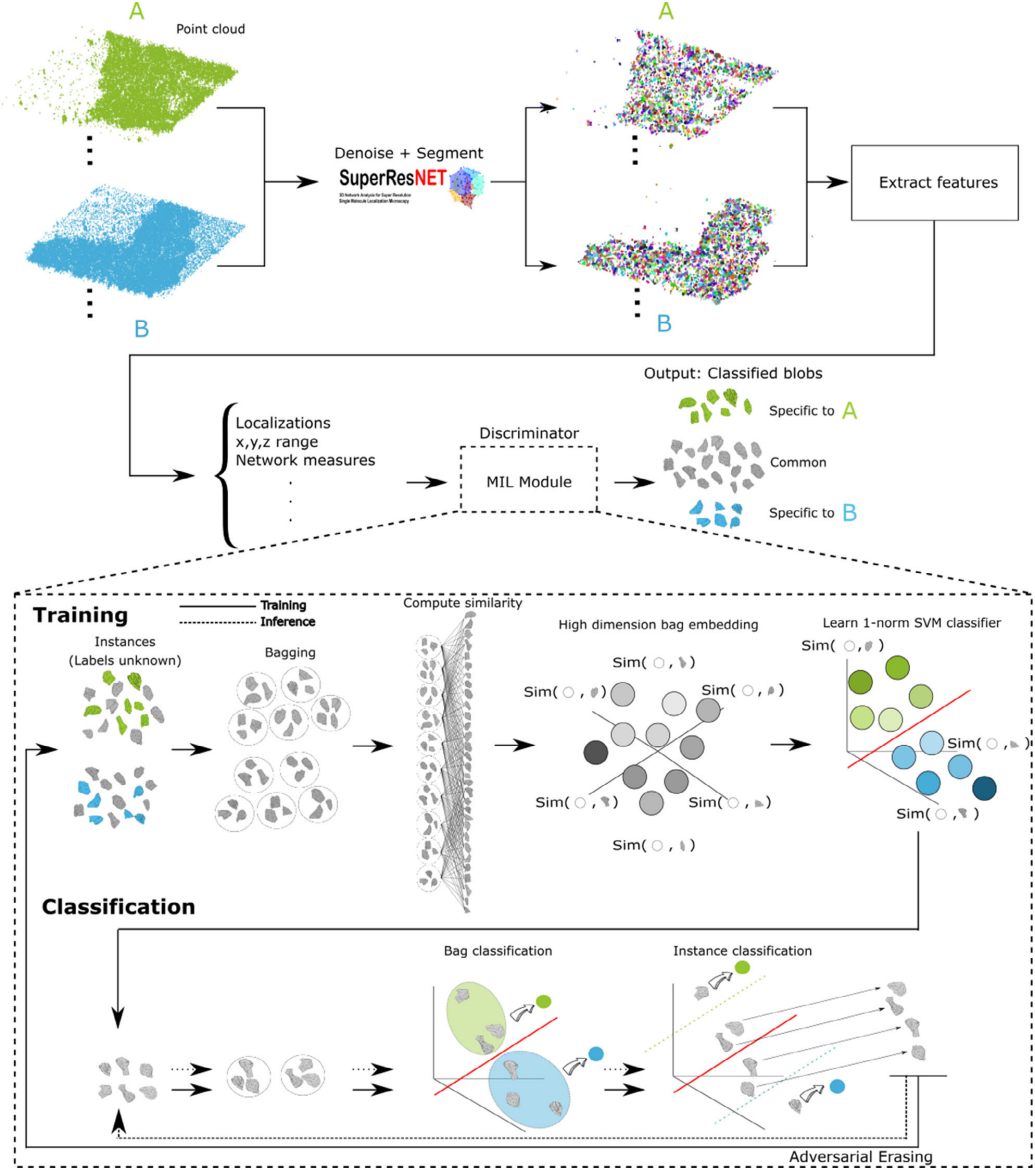
Overview of the proposed pipeline. The data preprocessing is shown at the top of the figure, which includes inputting SMLM conditions A and B into SuperResNET for denoising and segmentation, followed by feature extraction. Next, the processed data of both conditions are inputted into the siMILe module for classification into either structures specific to A, structures specific to B, or ambiguous. The details of siMILe are given within the dotted box. To train, the instances from a given class are grouped into bags, these bags are given an embedding by comparing all of its instances to the training data. Each dimension is represented by comparing the similarity of the bag to a given training instance. Next, a l1‐norm SVM classifier learns to fit a hyperplane that best separates the bags based on class. The instances of a bag can be labeled based on their contribution to the bag's classification. Unlabeled instances are reused in the adversarial erasing steps, where the model is retrained using them. Structures are visualized using the convex hull of its localizations for clarity.

In this article, not only are we the first to apply MIL to SMLM, but we enhance MIL via embedded instance selection (MILES) [23] for improved instance classification through the first use of adversarial erasing in MIL, enhanced with a new symmetric classifier (SC). Adversarial erasing iteratively removes identified structures and retrains the model, ensuring detection of all discriminative structures rather than just the most prominent ones. The SC enables simultaneous identification of structures unique to each condition in a single analysis, eliminating the need for separate comparisons and improving computational efficiency. siMILe, illustrated in Figure 1, uses only image‐level labels, allowing the potential for discovery by eliminating the need for manual annotations. The method is applied to a simulated dSTORM dataset to evaluate its efficacy and to PC3 and PC3‐CAVIN1 cells expressing cavin‐1 to display the ability of siMILe to extract discriminative structures from SMLM. The trained model from this dataset is then applied to a new dataset of PC3‐CAVIN1 cells dually labeled for Cav1 and cavin‐1 to investigate its generalizability and to verify using object level ground truth that the structures detected by siMILe are indeed discriminate from a biological perspective. This is achieved by validating the findings by contrasting them to protein interactions, information that was withheld from siMILe. We further extended the application of siMILe to clathrin‐mediated endocytosis, identifying structural changes in clathrin‐coated pits in response to small molecule inhibitors. These results support the ability of siMILe to identify changes in mesoscale domain structure, advancing our understanding of differential expression of intracellular molecular structures in distinct cell conditions.

## 2 Results

### 2.1 Problem Statement

In SMLM, each acquired image produces a 3D point cloud of molecular localizations. Throughout this paper, we use “image” and “point cloud” interchangeably to refer to these SMLM datasets. Formally, we are given input I: a set of n pairs $\left(P_{i},Y_{i}\right)$ for i=1,…,n, where $P_{i}$ is a point cloud and $Y_{i}$ is an associated class label. Each point cloud $P_{i}$ consists of (x,y,z) coordinates (3D vectors of real numbers), and the class labels $Y_{i}\in \{0,1\}$ indicate the condition from which the point cloud is derived.

Our expected output is a set of n pairs $\left(Q_{i},{\hat{Y}}_{i}\right)$ for i=1,…,n. Each $Q_{i}$ is a set of $J_{i}$ disjoint subsets $\left\{Q_{ij}:j=1,\ldots ,J_{i}\right\}$, where each subset $Q_{ij}\subset P_{i}$ represents a segmented structure and $Q_{ij}\cap Q_{ik}=\emptyset$ for all j≠k. Each ${\hat{Y}}_{i}$ is a set of labels $\left\{{\hat{Y}}_{ij}:j=1,\ldots ,J_{i}\right\}$ corresponding to the subsets, where ${\hat{Y}}_{ij}\in \{0,1,-1\}$ indicates whether subset $Q_{ij}$ is discriminative to condition 0, discriminative to condition 1, or common to both conditions (−1). Algorithm 1 summarizes the mathematical notation of the problem setup.

**Problem formulation for learning discriminative segmentations from point clouds with weakly supervised labels. The input is a set of 3D point clouds with a binary label for each point cloud based on its class. The output corresponding to a given input is a set of disjoint segmentations on the input, where each segmentation can be labeled as discriminative to the class given by the input label, or not discriminative (common).**

1 1: **Input**:2: $I=\{(P_{i},Y_{i})\;|\;1\leq i\leq n\}$ ▹ Input contains n point clouds each with a single label3: $P_{i}=\{P_{ij}\;|\;P_{ij}\in R^{3}\wedge 1\leq j\leq N_{i}\}$ ▹ Each point cloud $P_{i}$ is a set of $N_{i}$ real number 3D points.4: $Y_{i}\in \{0,1\}$ ▹ Label of point cloud $P_{i}$5: **Output**:6: $O=\{(Q_{i},\hat{Y_{i}})\;|\;1\leq i\leq n\}$ ▹ Output contains n new point cloud sets each with a set of labels7: $Q_{i}=\{Q_{ij}\;|\;Q_{ij}\subset P_{i}\wedge (Q_{ij}\cap Q_{ik}=\emptyset ,\;\forall k\neq j)\}$ ▹ Set of disjoint subsets of the input point clouds8: $\hat{Y_{i}}=\{{\hat{y}}_{ij}\;|\;{\hat{y}}_{ij}\in \{-1,0,1,\}\wedge 1\leq j\leq |Q_{i}|\}$ ▹ Subsets are given a class or null label

We use the SuperResNET network analysis platform to segment the point cloud, motivated by previous work supporting its ability to segment and extract clusters representing oligomeric biological structures [9, 11, 12]. A mean‐shift algorithm segments the localizations into clusters, constellations of 3D points, or ‘blobs’ that represent these structures. SuperResNET also extracts features from these blobs based on size, shape, topology, point statistics, and graph networks. We refer the interested reader for more detail to the original work [9]. After SuperResNET processes all point clouds from a given condition, it produces segmented structures called ‘blobs’ (the $Q_{ij}$ subsets). Each blob is represented by a 30‐dimensional feature vector, yielding a feature matrix of shape N×30 where N is the total number of blobs across all point clouds in that condition. We use this representation to compare conditions and extract blobs deemed distinct to their condition and are therefore discriminative. Next, we introduce MIL as the core underlying weakly‐supervised formulation used to learn the discriminative blobs in the output of our problem setup. We then introduce the diverse density (DD) MIL framework [24] that uses a single concept vector to classify bags of instances in feature space based on proximity to the concept vector. Next, we present multiple‐instance learning via embedded instance selection (MILES) [23] which extends DD with the use of multiple concept vectors. This enables learning more complex relationships for classifying bag labels, and provides an algorithm for blob classification. Finally, we detail our method, siMILe, which extends MILES. The contributions of siMILe focus on the integration of adversarial erasing (AE) for improved instance classification and a SC to reduce unnecessary computation time by asymmetric AE iterations. The base functionality of MIL algorithms is to identify discriminative from common objects, given two labels. But this can also cover the simpler use case where one set (A) or label is enclosed by the other one (B). In this case, there are no objects unique to A, only common to both and discriminative to B. In our problem statement, this case is not warranted; we need to be able to find those objects that are discriminative to each label individually. The combination of AE + SC in siMILe is designed to improve instance classification when discriminative instances exist in both classes (Figure 1).

### 2.2 MIL for SMLM

**FIGURE 2:**
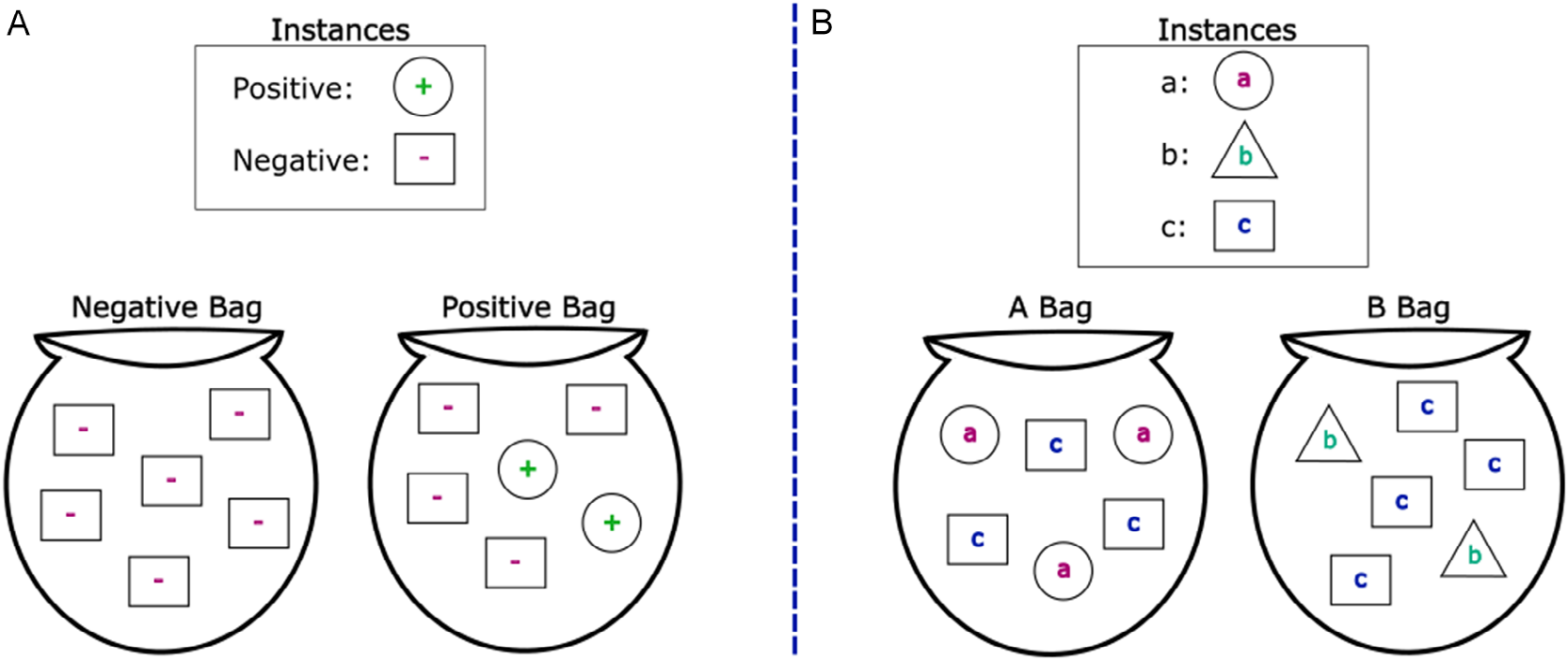
Visualizing two different multiple instance learning formulations. (A) Depicts the traditional MIL formulation with two classes: positive and negative. Both classes consist of negative instances, while only the positive class contains positive instances. Therefore, it is assumed that while a positive bag will have at least one positive instance, a negative bag will have only negative instances. (B) The case where both classes contain instances not found in the other. Class A contains instances of type a and c, while class B contains instances of type b and c. Thus, it is assumed that while each class contains instances shared with the other, they also contain instances that are specific to their class.

The problem formulation outlined in Algorithm 1 describes a weakly supervised learning paradigm where we need to extract discriminative blobs within an image using only the information provided by its condition label. Our method makes use of MIL, a weak supervision framework introduced by Dietterich et al. for drug activity prediction [25]. MIL identifies objects or “instances” under conditions where class labels are represented by sets of instances, as opposed to individual instances. In the context of this article, these “instances” are the blobs (segmented structures) produced by SuperResNET, representing approximate protein structures. Training data points (the instances/blobs in our application) are grouped into bags containing a set number of instances. Using these bags, where each bag has a label that corresponds to a condition label under which the image is acquired, the focus of some MIL methods (e.g., MI‐support vector machine (SVM) [26], Citation‐kNN [27], EM‐DD [28]) is predicting a bag label using the aggregated information of its instances. For other methods (e.g., mi‐SVM [26], APR [25]), it is more important to classify the individual instances by taking advantage of the representations learned by predicting their bag. Aggregating the information of the instances is useful when some instances are not capable of representing their weak label, while others are, and this distinction is unknown. In the traditional MIL formulation, there are two bag classes: positive and negative. The positive class contains instances that can be labeled as positive or negative, whereas the negative class is assumed to only contain negative instances. This 2‐class setup is visualized in Figure 2A. Given that an instance of the positive class has the potential to be found in the negative class, classification is performed on bags. In positive bags, the proportion of positive instances it contains is referred to as the witness rate [29]. In the traditional formulation, it is assumed that there is at least one positive instance in the bag; otherwise, a positive bag would be indistinguishable from a negative bag.

### 2.3 MILES

**FIGURE 3:**
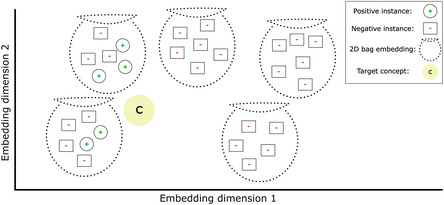
DD concept demonstrated. Example positive bags (green) and negative bags (red) are embedded into 2D and plotted. A concept capable of differentiating the positive and negative bags is also plotted in the same space. The positive bags have a closer distance to the concept than the negative bags, meaning that there exists a threshold on this distance capable of correctly labeling all bags.

DD [30] is a MIL algorithm notable for the use of a single “concept” to aid in classifying bags, where this concept is a *representation of the relationship between positive and negative classes*. The target concept is assumed to be closer to positive instances and further away from negative ones, providing a method to differentiate the bags according to their label, as seen in Figure 3. Given a potential concept c, positive bag $B_{i}^{+}$, and negative bag $B_{i}^{-}$, the concept that is most likely to fit the bags is determined by the number of positive and negative bags that agree on the concept by maximizing

(1) $$\begin{array}{cc} & DD(c)=\prod_{i}Pr(c|B_{i}^{+})\prod_{i}Pr(c|B_{i}^{-}) \end{array}$$

Given that $b_{ij}^{+}$ is an instance in bag $B_{i}^{+}$, and $b_{ij}^{-}$ is an instance in bag $B_{i}^{-}$, a proposed method to estimate $Pr(c|B_{i})$ is the following:

(2) $$\begin{array}{cc} & Pr(c|B_{i}^{+})\propto \max_{j}\exp\left(\frac{-{\left\|b_{ij}^{+}-c\right\|}^{2}}{\sigma^{2}}\right) \end{array}$$

(3) $$\begin{array}{cc} & Pr(c|B_{i}^{-})\propto 1-\max_{j}\exp\left(\frac{-{\left\|b_{ij}^{-}-c\right\|}^{2}}{\sigma^{2}}\right) \end{array}$$

Equation (2) finds the closest distance between any instance in the positive bag $B_{i}^{+}$ to the concept, maximizing the value towards 1 as the distance decreases. Similarly, Equation (3) finds the same distance but reaches the maximum value as the distance increases instead. The distance between an instance and the concept is scaled by $\frac{1}{\sigma^{2}}$. When the scaling factor σ is small, an instance needs to be closer to the concept to achieve high similarity.

Multiple‐Instance learning via embedded instance selection (MILES) extends the use of a single target concept to the use of many target concepts. With this change, it is now assumed that the ensemble of concepts is capable of representing more complex relationships between the bags than those possible by a single concept that is required to be closer to positive bags. For example, a given concept may instead be closer to the negative bags than to the positive bags. Given a set of concepts C, the estimate of $Pr(c|B_{i})$ is now defined independently of the bag label

(4) $$\begin{array}{cc} & Pr(c|B_{i})\propto \max_{j}\exp\left(\frac{-{\left\|b_{ij}-c\right\|}^{2}}{\sigma^{2}}\right),c\in C \end{array}$$

Given $N=N^{+}+N^{-}$ where $N^{+}$ is the number of instances of the positive class and $N^{-}$ is the number of instances of the negative class, MILES uses all N instances as potential concepts; $C=\{c_{1},c_{2},\ldots ,c_{N}\}$. If each instance in a bag $B_{i}$ is represented by a single feature vector $b_{ij}$, then $B_{i}$ can be embedded in a N‐dimension vector $E_{i}$ through an aggregation of its instances as follows

(5) $$\begin{array}{c} E_{i}={\left[sim(B_{i},c_{1}),sim(B_{i},c_{2}),\ldots ,sim(B_{i},c_{N})\right]}^{T} \end{array}$$

(6) $$\begin{array}{c} sim(B_{i},c)=\max_{j}\exp\left(\frac{-{\left\|b_{ij}-c\right\|}^{2}}{\sigma^{2}}\right) \end{array}$$

The value of $E_{ik}$, k∈{1,2,…,N} is equal to the similarity between the most similar instance in $B_{i}$ to the concept $c_{k}$ as defined in Equation (6).

Given all our bags, represented by their embedding vector and a bag label, the algorithm learns to classify the bags through an L1‐norm linear SVM [31]. The SVM learns to fit a hyperplane that best separates the bags based on their label through an optimization process, where the separation is performed in the same space as the bags’ embedding. Since an L1‐norm penalty is applied, the optimization process is incentivized to reduce the sum of the absolute value of the hyperplane weights, leading to sparsity. A bag is simply classified on the basis of the side of the SVM hyperplane it resides.

Each dimension in the embedding of a bag is based on the most similar of its instances to a concept, but this concept can be equally similar to multiple instances. For a concept $c_{k}$, the number of these instances is $m_{k}$. From the perspective of an instance $b_{ij}$, there is also a set of concepts to which it was among the most similar in bag $B_{i}$ to; we denote the set of indices corresponding to these concepts as $I_{j}$. Given that $w_{k}$ is the $k^{th}$ weight in the SVM hyperplane, the classification score of an instance is

(7) $$\begin{array}{c} ICS(b_{ij})=\sum_{k\in I_{j}}\frac{w_{k}sim(B_{i},c_{k})}{m_{k}} \end{array}$$

The ICS of $b_{ij}$ is computed by summing all similarity values that $b_{ij}$ contributed to the bag embedding, with each value scaled by its corresponding SVM weight $w_{k}$ and normalized by $m_{k}$, the number of instances equally similar to concept $c_{k}$. In this step, the required use of a linear SVM is seen, where the weights of the SVM hyperplane can be directly mapped to the features of the bags embeddings. Once the classification score of an instance is calculated, a threshold is used to determine its label. Although the authors of MILES acknowledge that different thresholds may be used, they recommend that anything greater than $\frac{-b}{\left|U_{i}\right|}$ be labeled positive. Where b is the SVM hyperplane bias, and $U_{i}$ is the minimal set of instances in $B_{i}$ required to create its embedding as defined in Equation (5).

### 2.4 SiMILe

Although MILES is powerful, only a single positive instance is theoretically required to correctly classify a bag. This means that a bag containing multiple positive instances can ignore some of them during the creation of the bag embedding or during the SVM learning phase, while still maintaining equivalent performance on bag classification. This susceptibility to using only a fraction of the discriminative features to classify is a problem intrinsic to many such classifiers, hindering MILES’ ability to correctly classify all positive instances. Furthermore, as described, MILES assumes a traditional MIL formulation, making it inefficient when the goal is to compare two classes and extract discriminative instances from each, instead of from only one. For example, as given in Figure 2B, assume you have classes A and B, where class A contains instances a and instances c, while class B contains instances b and instances c. When looking to predict the discriminative instances in these classes, which are the instances a and b, MILES would require two training phases. In the first phase, we would declare class A the positive class and label all instances in class B negative, since instances a are now considered positive instances; MILES would look to label them as positive. During the second run, the classes are swapped and the B class is considered positive; the same procedure is used, except with the goal of applying positive labels to instances b. This requirement of multiple phases is inefficient and can cause unstable results in the case that the model learns by focusing only on the discriminative instances that exist within the negative class. In this case, positive bags are classified as positive based only on the absence of discriminative instances found in the negative bags, which means that the difference between positive and negative instances in positive bags is not represented in either the bag embedding or the learned SVM weights, to the detriment of instance classification.

**FIGURE 4:**
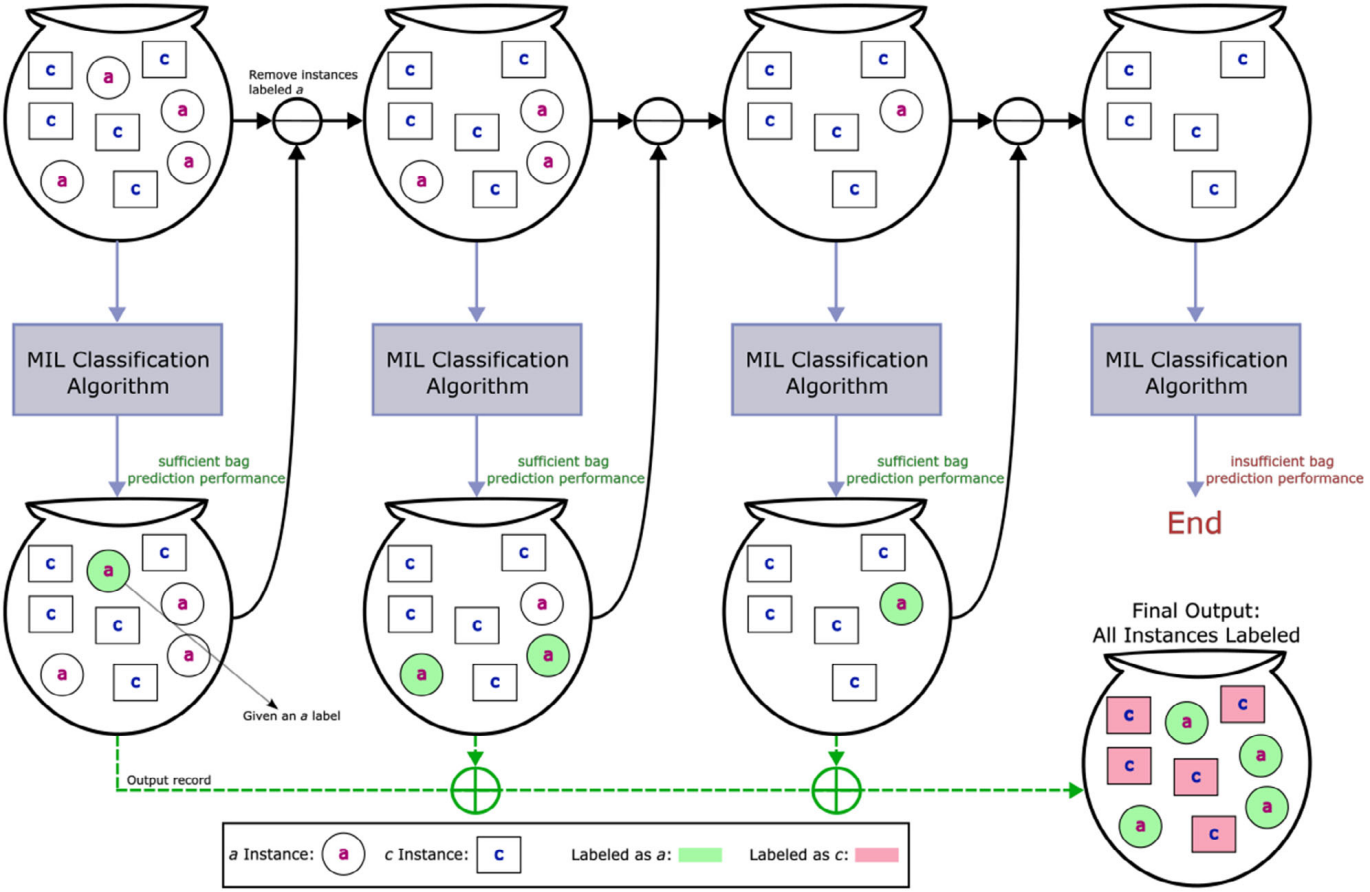
Adversarial erasing applied to MIL. The visual is given in the context of the formulation where two classes are compared; A and B, with class A having instances a and c, while class B has instances b and c. The goal in this formulation is to label the instances a and instances b. The visualization follows the state of a single class A bag through adversarial erasing iterations. In each iteration the classifier is trained to separate bags by class, after which it applies labels to the instances in the bag. All instances given a label a are removed from the bag before the next iteration. The iterations stop when attempting to train the classifier no longer results in the ability to sufficiently classify bags, at which point all the labels given through the iterations are collected, with the remaining instances labeled as c. The instances labeled a in early iterations are those that were the focus of the classifier while training at that iteration, by removing them before the next iteration, the classifier is forced to learn based on the remaining.

To alleviate the problem caused by the use of a subset of discriminative positive instances to classify, we improve MILES by implementing an adversarial erasing scheme (Figure 4). In adversarial erasing, the goal is to extract all discriminative instances by training the model over multiple iterations and removing the predicted discriminative instances before the next iteration. In the traditional MIL case, this would mean removing positive instances and retraining the classifier until no more positive instances can be found. The iterations will end when the performance of the bag classifier is deemed insufficient. Practically, we set a minimum accuracy in bag classification performance, denoted minacc, to determine when to stop further iterations. Adversarial erasing also helps in the situation where the classifier focuses only on the discriminative instances in one class. Should this happen, those instances would be iteratively removed until the classifier is forced to look at the discriminative instances in the other class.

To optimize the algorithm to classify both classes in a single run, we replace the single threshold classification of the instance classification scores with a k‐means scheme using three centers. Given the set of all predicted instances as X, a given instance $b_{ij}\in X$ is predicted as follows

(8) $$\begin{array}{c} V=\{ICS(x):x\in X\} \end{array}$$

(9) $$\begin{array}{c} t_{A},t_{null},t_{B}=kmeans_{3}(V) \end{array}$$

(10) $$\begin{array}{c} c_{ij}=arg\;min_{t\in \{t_{A},t_{null},t_{B}\}}\left(\left|ICS(b_{ij})-t\right|\right) \end{array}$$

(11) $$\begin{array}{c} y_{ij}=\left\{ \begin{array}{cc} 1 & c_{ij}=t_{A}\\ -1 & c_{ij}=t_{null}\\ 0 & c_{ij}=t_{B} \end{array}\right. \end{array}$$

The scores of all predicted instances are aggregated and used with k‐means to find three class centers. The label of a given instance is applied based on the center it was closest to. Using this SC to classify discriminative instances in both classes, siMILe avoids the computational cost of running the asymmetric base algorithm twice. Furthermore, through the use of clusters, the algorithm is able to be more conservative in its labeling, only labeling instances that cluster around the discriminative centers rather than labeling all instances above a fixed threshold as in traditional MILES. While this could normally be to the detriment of the algorithm's ability to classify all discriminative instances, the use of adversarial erasing iterations negates this risk and instead enables more precise instance classification (Section 2.5).

While it is in principle possible to use deep learning for the classification step, we select an SVM as the basis for training in siMILe to keep training time very short. Although adversarial erasing is powerful in its ability to label more discriminative instances and enable the use of MIL on classes that each contain these discriminative instances, its iterative retraining could become prohibitive when using a deep learning architecture instead. This is particularly seen in cases where these iterations can become numerous. Future work can consider extending this to methods in which retraining can be done using deep learning approaches without retraining from scratch, but this is beyond the scope of this work. By leveraging an SVM, siMILe maintains a relatively low training time, enabling use on less powerful hardware, while also not requiring a graphics processing unit and keeping siMILe's carbon footprint sufficiently small. Our results (Section 2.5 and onward) show that performance is not meaningfully compromised by these choices.

### 2.5 Evaluation on Simulated Dataset

**FIGURE 5:**
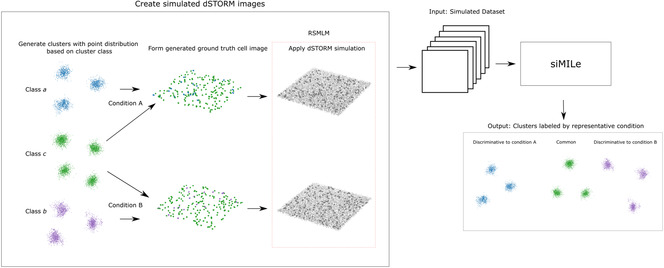
Generating the two conditions of the simulated dSTORM dataset and applying siMILe to identify condition‐specific clusters. The simulated dSTORM dataset consists of 3D point clouds, each labeled as condition A or B. Condition labels are determined by the cluster classes present within the point clouds, which differ by their generative distributions. Specifically, condition A contains clusters from classes a and c, while condition B includes clusters from classes b and c. These point clouds are processed with the RSMLM [32] package to simulate dSTORM image acquisition. Once multiple datasets for each condition are generated, the siMILe pipeline is applied to identify and label clusters as either condition‐specific (discriminative) or common to both conditions.

**FIGURE 6:**
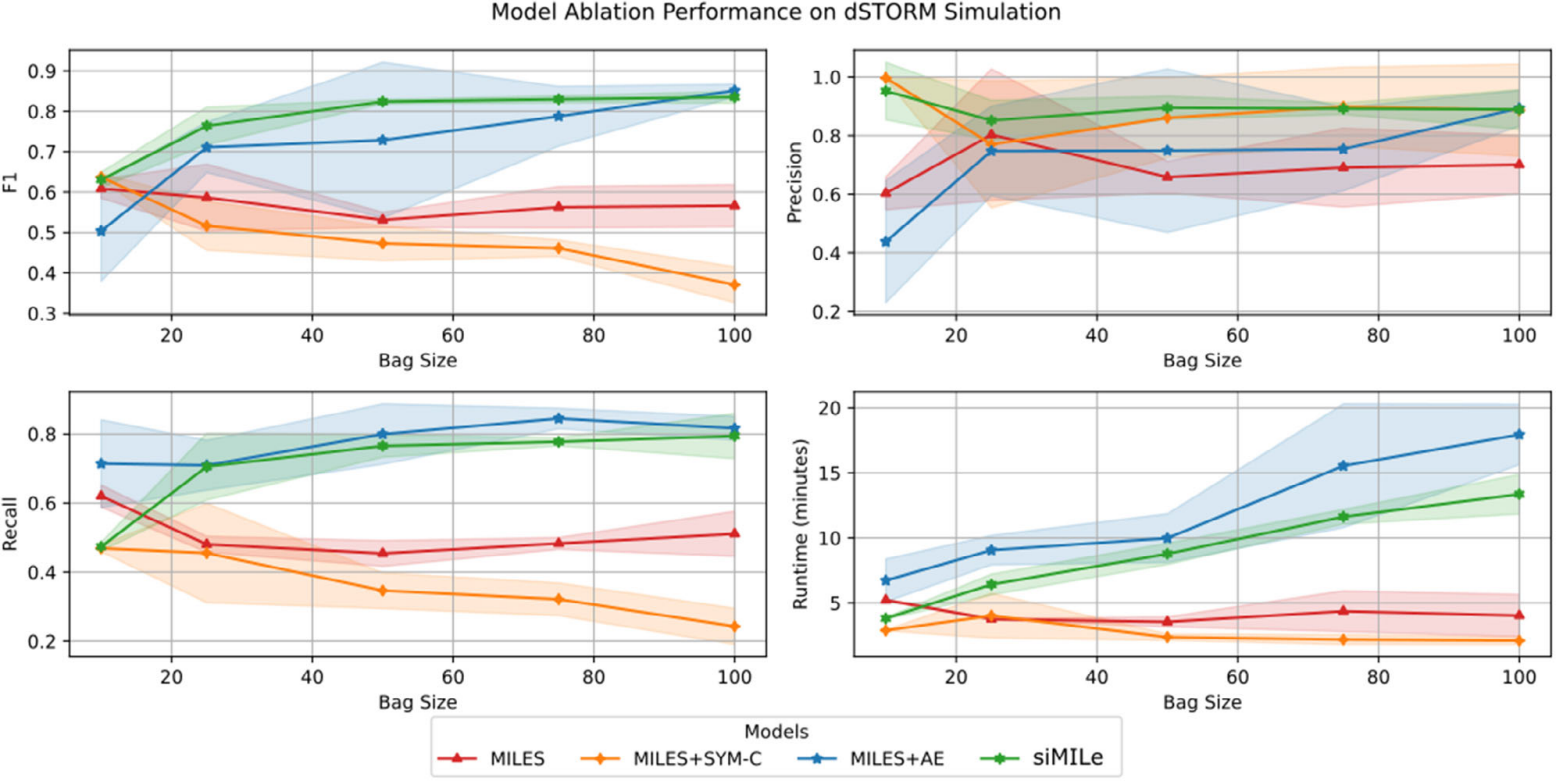
Ablation results on instance classification performance and single core runtime. The highest F1 score is maintained by siMILe across bag sizes, followed closely by MILES + AE, then MILES, with MILES + SYM‐C having the worst performance. The recall is similiar to F1 except for MILES + AE performing slightly better than siMILe regardless of bag size. The highest precision scores are siMILe and MILES + SYM‐C while MILES has the lowest except as smaller bag sizes. The models using AE have significantly higher training times, increasing with bag size when maximizing classification performance, while MILES and MILES + SYM‐C maintain low train times regardless of bag size when achieving their best classification performance. Lines show mean values from nested 5‐fold cross‐validation with shaded regions indicating ±1 standard deviation.

We first detail the performance of siMILe on simulated data to quantify the effect of our algorithmic improvements between siMILe and MILES. Simulated SMLM data enables us to have full control of the objects and noise model in the data, making it ideal to validate our stated claims that differentiate siMILe from MILES. Using the RSMLM [32] package to simulate dSTORM image acquisition, we generated three types or classes of clustered localizations, or “blobs”: A, B, C. We grouped them in two labels or “conditions”: Sets with label A containing clusters of type a and c, while sets with label B contain clusters of type b and c (Figure 5). We compare the ability of siMILe to extract discriminative instances from each class against MILES, MILES with adversarial erasing (MILES + AE), and MILES with the SC (MILES + SYM‐C). We report the comparison metrics relative to bag size to alleviate the potential issue of specific algorithms having better computational performance based on bag size. The results are also provided based on the average of a nested cross‐validation using 5 folds, with the standard deviation reported. The results for F1, precision, recall, and training time are given in Figure 6. Figure 6 shows that siMILe outperforms MILES and the isolated improvements such as SYM‐C and AE consistently across bag sizes in all metrics. The exception occurs for very small bag sizes, but as the reader can confirm, here all results for all methods are too noisy to extract consistent patterns.

The training time of a model is recorded using an Intel Core i7‐7740X with 32 GB RAM available on a machine running Ubuntu 20.04; the time is reported based on use of a single core. Models using AE include all iterations in their recorded runtime. Runtime results show that siMILe has quite short training times (<20 min), enabling users to retrain it quickly if needed. siMILe has an increased training time compared to MILES, trading training time for improved performance. Given the low overall training time, this is a worthwhile compromise.

### 2.6 SiMILe Identifies Caveolae as Discriminative Structures between PC3 and PC3‐CAVIN1 Cells

Next, we evaluate siMILe on its capability to detect complex discriminative protein structures in real dSTORM data using the PC3/PC3‐CAVIN1 dataset [9]. Considering that Cav1 does not form caveolae in the absence of cavin‐1, we investigate the ability of siMILe to detect caveolae in cavin‐1‐expressing PC3 cells (referred to as PC3‐CAVIN1) in comparison to PC3 cells lacking cavin‐1 and not presenting caveolae [33].

**FIGURE 7:**
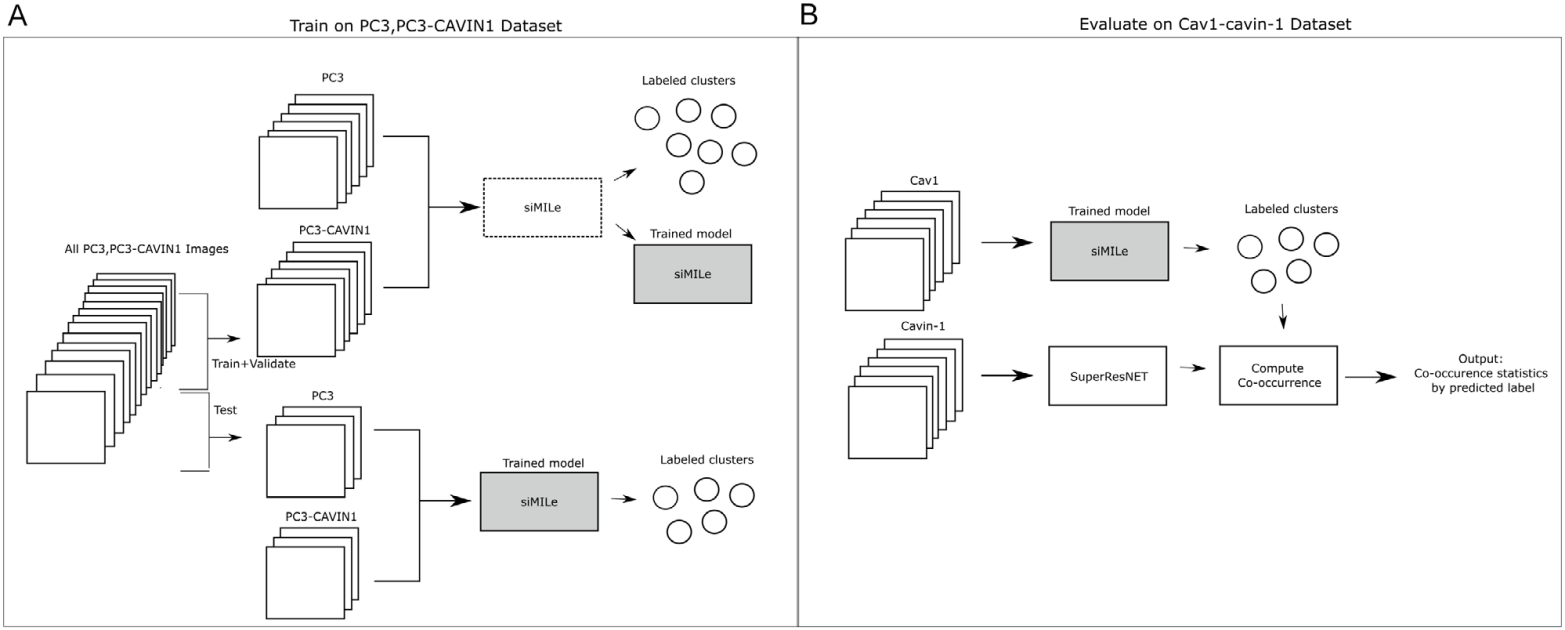
Experiment setup for the application of siMILe to PC3,PC3‐CAVIN1 and two‐channel Cav1‐cavin‐1 datasets. (A) The PC3,PC3‐CAVIN1 3D point cloud dataset is split into training, validation, and testing subsets. The training and validation sets are used to train siMILe and fine‐tune hyperparameters for labeling clusters. The final trained model is applied to the test set to generate the reported results. (B) The pretrained model from A is applied to the Cav1 channel of the Cav1‐cavin‐1 dataset. Clusters identified by the model are labeled and compared against the SuperResNET‐processed cavin‐1 channel to assess cooccurrence by label.

**FIGURE 8:**
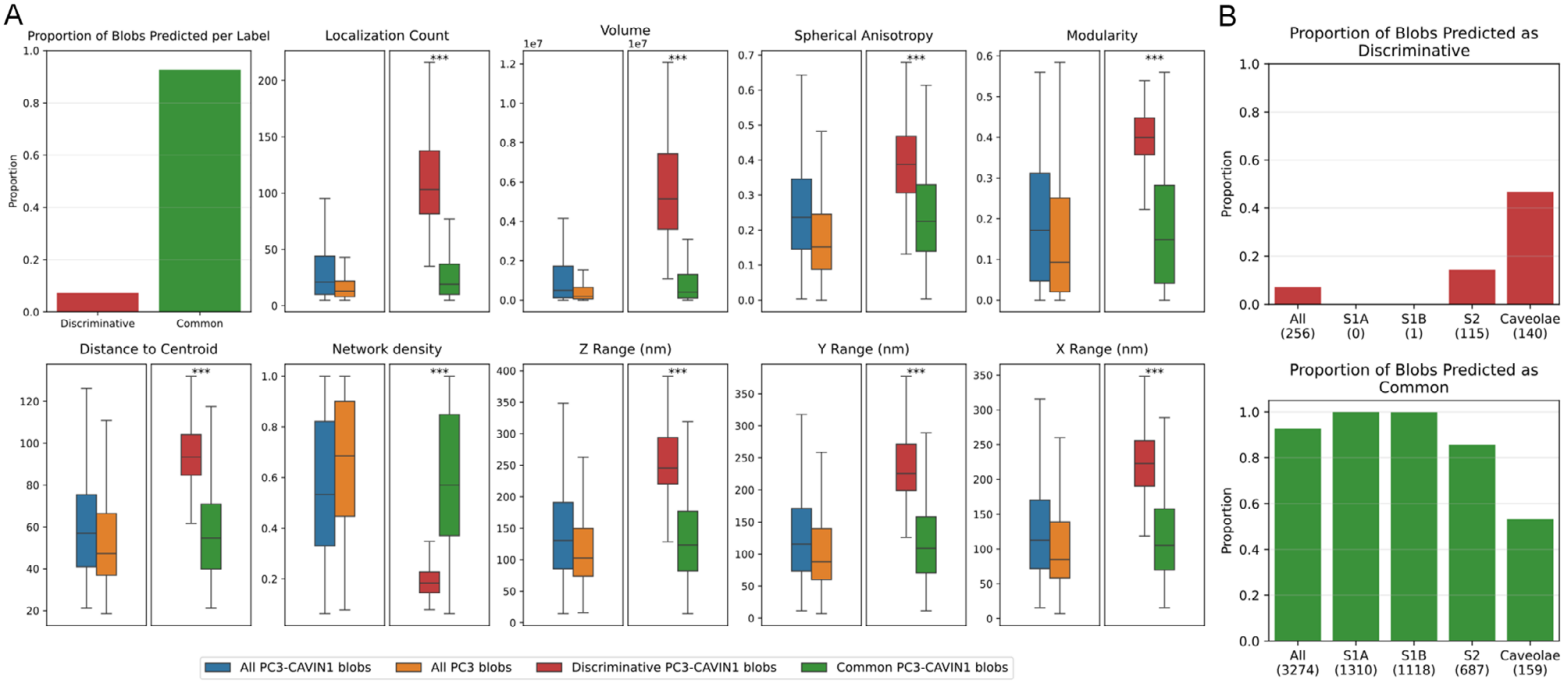
Feature comparison of predicted blob labels in the PC3,PC3‐CAVIN1 dataset. After applying siMILe to compare the PC3 and PC3‐CAVIN1 conditions, we show the proportion of labels predicted in the first plot out of the 7060 blobs, with ∼93% of the blobs labeled as common. The feature distribution of these blobs, based on the predicted labels (discriminative in red and common in green), is compared against the feature distribution of all blobs in the PC3 (orange) and PC3‐CAVIN1 (blue) datasets. The discriminative blobs are consistently larger in size with many more localizations. They also appear to be more spherical, contain less dense networks, and contain more modules. These features are signature for caveolae, one of the known unique differences between PC3 and PC3‐CAVIN1. This shows that siMILe is able to isolate caveolae as unique to PC3‐CAVIN1, given that they require cavin‐1 to form. Objects identified as caveolae in panel B are classified using SuperResNET. It is possible that SuperResNET mislabels some S2 as caveolae and vice versa, which can explain the partial agreement of siMILe and SuperResNET. Box plots show median (center line), interquartile range (box), and 1.5× IQR (whiskers). Statistical comparisons were performed using the two‐sided Mann–Whitney U test. Significance levels: 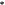 *p* < 0.05, 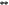 *p* < 0.01, 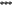 *p* < 0.001. Nonsignificant comparisons (*p* ≥ 0.05) are not marked.

Using the features produced by SuperResNET, we apply siMILe to classify the clusters in PC3‐CAVIN1 as discriminative to PC3‐CAVIN1 or common to PC3 (not discriminative) (Figure 7). Our results labeled 8% of blobs within PC3‐CAVIN1 as discriminative (Figure 8). Blobs labeled discriminative to PC3‐CAVIN1 cells are more spherical and much larger than the common blobs. SuperResNET classification (which categorizes Cav1 structures as S1A scaffolds/8S complexes, S1B and S2 scaffolds as intermediate oligomers or caveolae based on size and morphology) of the discriminative blobs shows that they consist predominantly of caveolae, with a smaller proportion of S2 scaffolds, 1 S1B scaffold and no S1A scaffolds (Figure 8B). This is consistent with the 200–250 nm size and 90 nm distance to centroid of the point clouds, as well as localization counts approaching that of the estimated 144±39 in caveolae [34]. It is clear that the model is identifying caveolae‐like structures as discriminative to PC3‐CAVIN1.

### 2.7 Transfer of Trained Model to Dual‐Channel Cav1‐CAVIN‐1 Dataset

**FIGURE 9:**
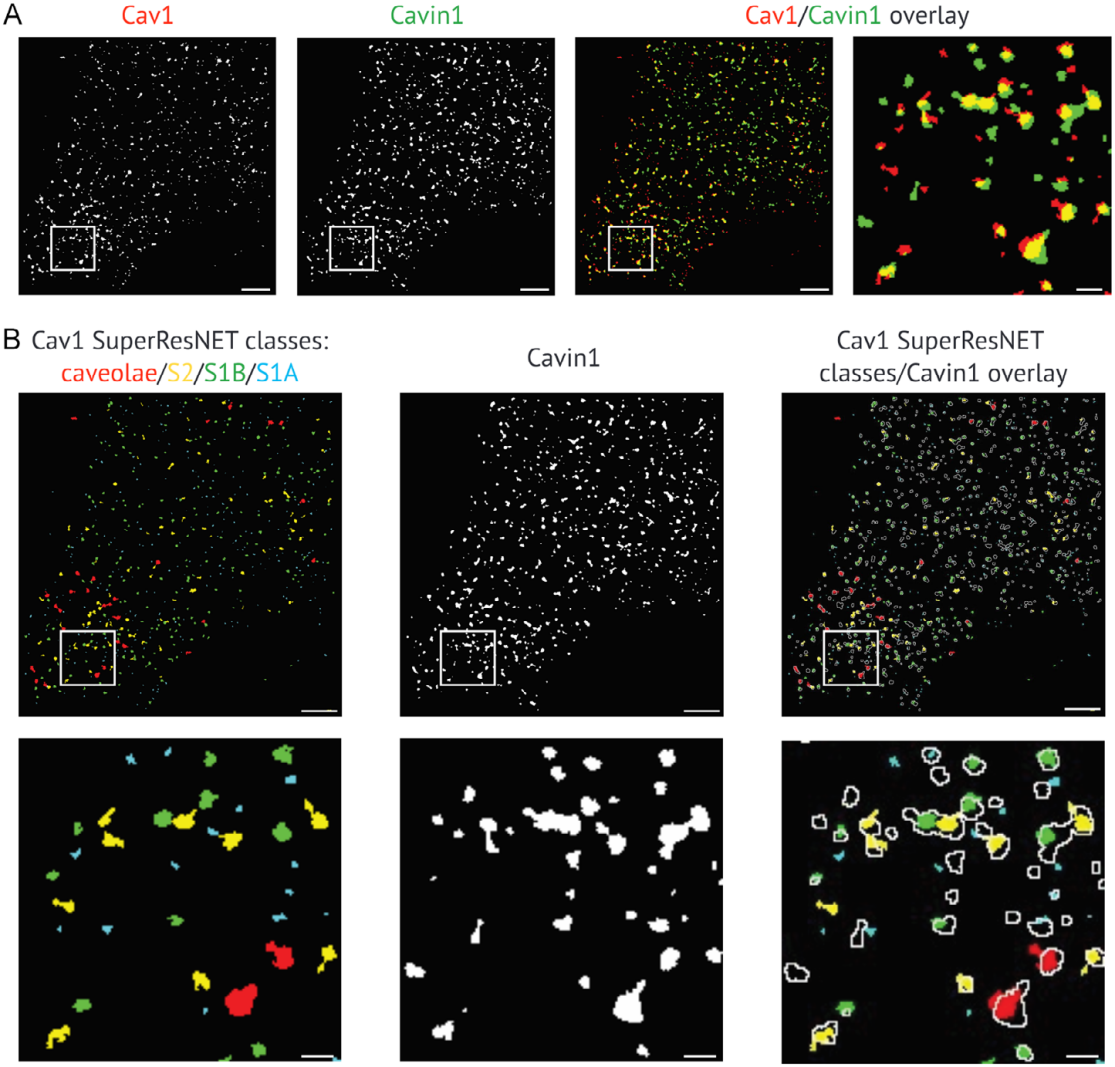
SuperResNET classification of two‐channel Cav1‐cavin‐1 dSTORM images. (A) 2D mask overlay of 3D Cav1‐cavin‐1 two‐channel dSTORM images. Scale bars: 2 μm (whole image) and 150 nm (inset). (B) Overlay of Cav1 SuperResNET classes (red: caveolae, yellow: S2, green: S1B, cyan: S1A) and cavin‐1. Scale bars: 3 μm (whole image) and 250 nm (inset).

Having identified discriminative structures in PC3‐CAVIN1 cells, we next sought to validate these findings using an independent dataset with biological ground truth. We applied the trained model to PC3‐CAVIN1 cells dually labeled for both Cav1 and cavin‐1, where cavin‐1 colocalization provides independent validation of caveolar structures. The Cav1 channel was processed by SuperResNET to identify S1A, S1B, and S2 scaffolds as well as caveolae [9] and then overlaid with the cavin‐1 labeled channel. Representative two‐channel dSTORM images show the spatial distribution of Cav1 and cavin‐1 in PC3‐CAVIN1 cells, with SuperResNET classification revealing the organization of different Cav1 structural classes relative to cavin‐1 (Figure 9).

**FIGURE 10:**
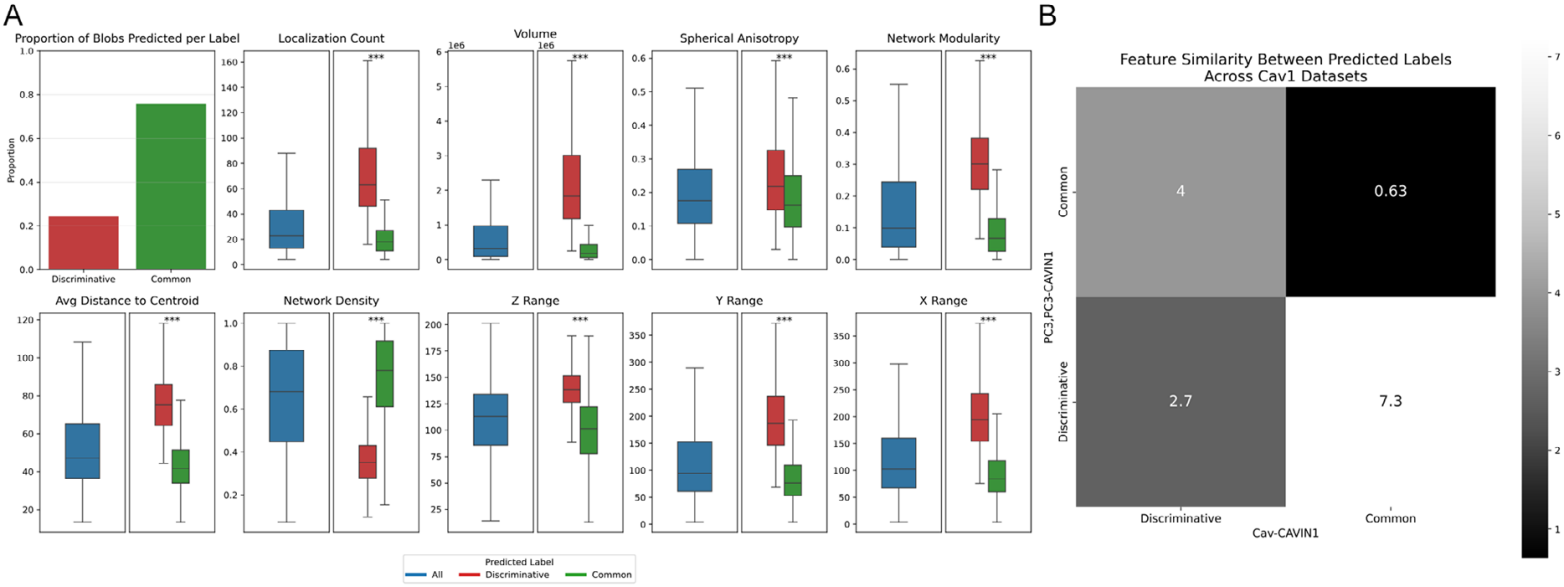
Feature comparison of predicted blob labels in the Cav1‐cavin‐1 dataset from trained model. (A) After applying siMILe trained on PC3,PC3‐CAVIN1 to the Cav1 channel of the Cav1‐cavin‐1 dataset, the proportion of labels is shown in the first plot, with the majority of the 30,412 blobs labeled as common. The feature distribution of these blobs, based on the predicted labels (discriminative in red and common in green), is compared against the feature distribution of all blobs in the Cav1 (blue) images. The discriminative blobs consistently follow the trend seen in the PC3‐CAVIN1 predictions. These blobs are of a larger size, contain a higher count of localizations, are more spherical, have less dense networks, and have more modules. (B) The predicted labels are compared between both datasets through a normalized euclidean distance, supporting the similarity of these labels across both datasets. Box plots show median (center line), interquartile range (box), and 1.5× IQR (whiskers). Statistical comparisons were performed using the two‐sided Mann–Whitney U test. Significance levels: 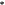 *p* < 0.05, 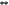 *p* < 0.01, 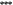 *p* < 0.001. Nonsignificant comparisons (*p* ≥ 0.05) are not marked.

Application of the PC3/PC3‐CAVIN1 trained model to the Cav1 channel of this new dataset identified 22% of blobs as discriminative (Figure 10A). Feature distributions of discriminative versus common blobs showed similar patterns to those observed in the original dataset, with discriminative blobs exhibiting larger size, higher localization counts, increased sphericity, less dense networks, and more modules. The normalized Euclidean distance between blob classes in both datasets confirmed high feature similarity (Figure 10B), demonstrating successful model transfer.

### 2.8 Cavin‐1 Interaction Validates Discriminative Structure Identification

To determine whether discriminative structures identified by siMILe represent biologically relevant populations, we assessed their association with cavin‐1. To quantify the spatial proximity between Cav1 and cavin‐1 structures, we calculated a Blob Overlap Parameter (BOP) for each Cav1 blob as $-\log(d/(r_{\text{cav1}}+r_{\text{cavin‐1}}))$, where d is the distance between the Cav1 blob centroid and its nearest cavin‐1 blob centroid, and r represents the respective radii. Thus, a BOP value of 0 indicates structures with touching borders, positive values indicate overlapping structures with higher values representing greater overlap, and negative values indicate separated structures with more negative values representing greater separation.

**FIGURE 11:**
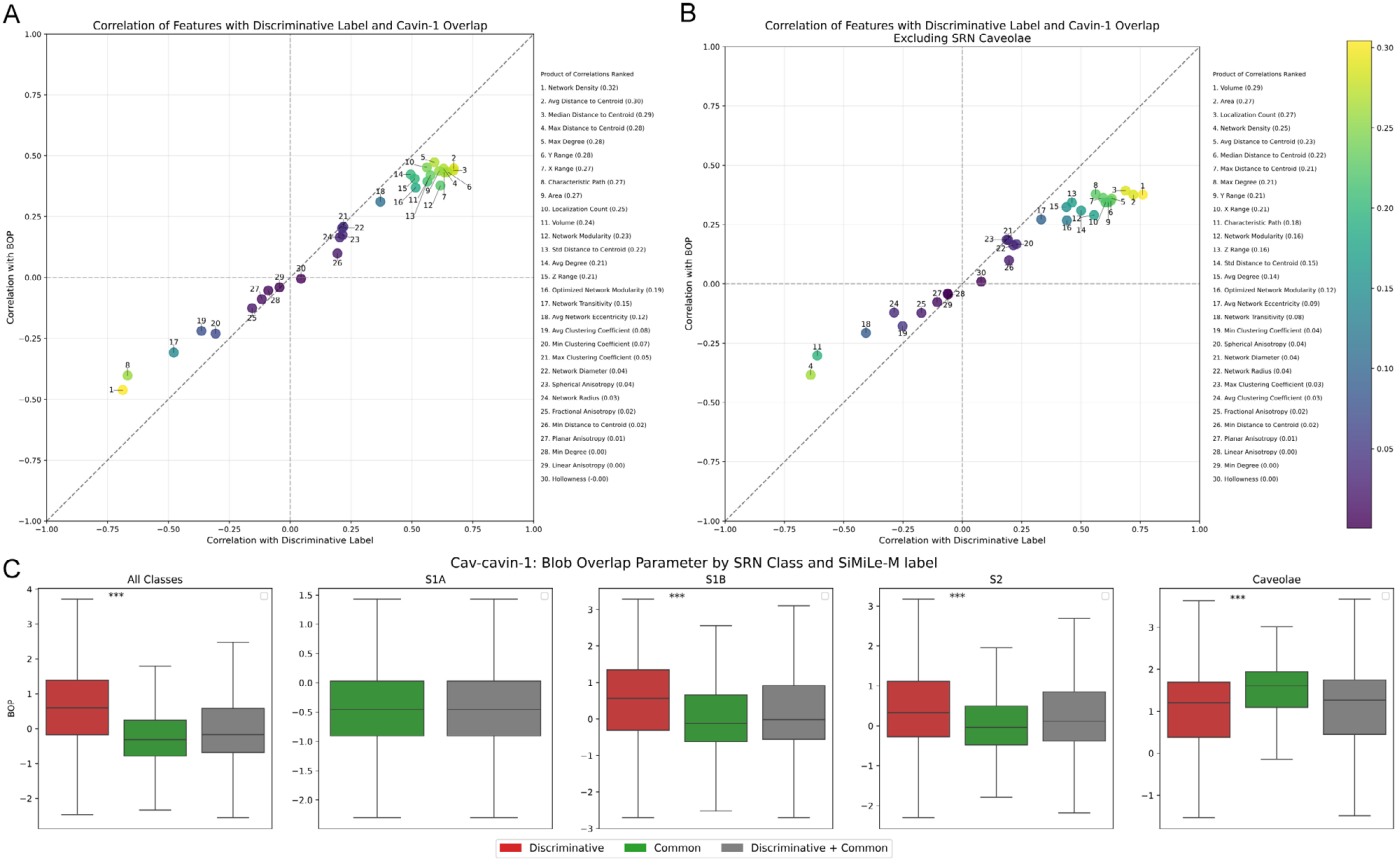
Correlation analysis of network features with discriminative labels and cavin‐1 interaction. (A) Scatter plot showing the correlation of blob features with the discriminative label (*x*‐axis) and cavin‐1 interaction (*y*‐axis). Features follow a positive correlation trend, indicating a consistent relationship between discriminative properties and increased cavin‐1 interaction. The product of correlations ranked (right) highlights network density, distances to centroid, and spatial range parameters as top contributors. (B) Similar correlation analysis excluding SRN caveolae samples. The exclusion slightly alters correlation rankings, with volume, area, localization count, and network density being the strongest contributors. (C) Box plots displaying cavin‐1 Blob Overlap Parameter (BOP) distributions across different SRN classes (All Classes, S1A, S1B, S2, and caveolae), separated by siMILe predicted labels (discriminative in red, common in green, combined in gray). Across SRN S2 and S1B, discriminative blobs exhibit larger overlap compared to common blobs based on the larger BOP values, indicating that siMILe identifies structures with cavin‐1 association patterns despite not being SRN caveolae. Box plots show median (center line), interquartile range (box), and 1.5× IQR (whiskers). Statistical comparisons were performed using the two‐sided Mann–Whitney U test. Significance levels: 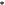 *p* < 0.05, 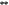 *p* < 0.01, 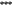 *p* < 0.001. Nonsignificant comparisons (*p* ≥ 0.05) are not marked.

Correlation analysis revealed that features associated with discriminative labeling also correlated with cavin‐1 interaction (Figure 11A, all correlations *p* < 0.001). Network density showed the strongest correlation product (0.32), with both discriminative and cavin‐1‐interacting structures exhibiting lower density values. Distance to centroid metrics all correlated positively with both parameters, yielding correlation products of 0.30 (average), 0.29 (median), and 0.28 (maximum). Spatial parameters (Y range: 0.28, X range: 0.27), structural features (max degree: 0.28, area: 0.27), and network modularity (0.23) also showed positive correlations with both discriminative labeling and cavin‐1 interaction. Characteristic path length showed negative correlations with both parameters (correlation product: 0.27).

Analysis excluding SuperResNET‐classified caveolae showed similar correlation patterns for scaffold structures (Figure 11B, all correlations *p* <0.001). Size‐related features showed the highest correlation products, with volume (0.29), area (0.27), and localization count (0.27) all positively correlating with both discriminative labeling and cavin‐1 interaction. Network density maintained negative correlations with both parameters (0.25), while distance to centroid metrics showed positive correlations (0.21–0.23). Network modularity showed positive correlations (0.16), though reduced compared to the full dataset.

**FIGURE 12:**
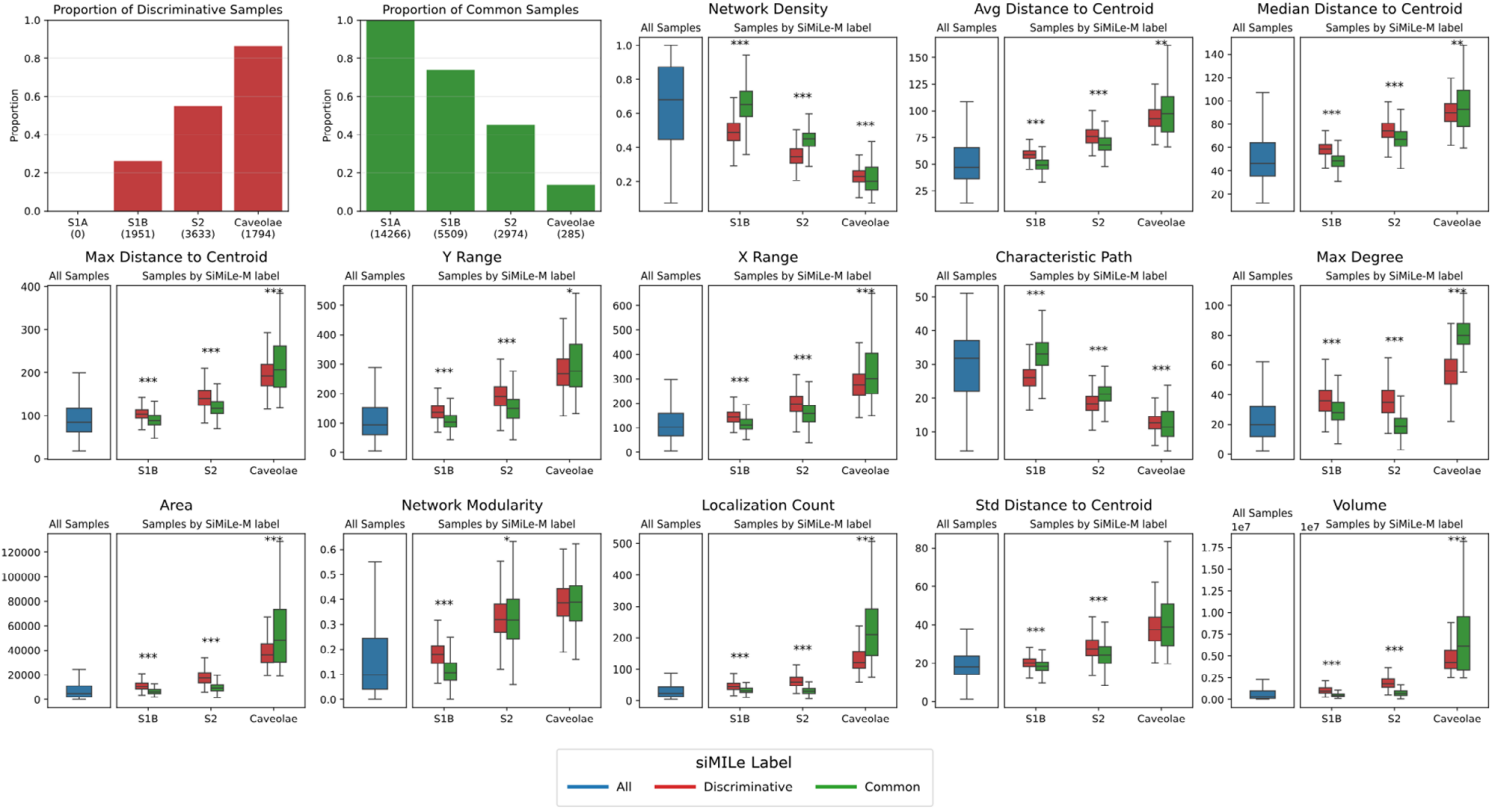
Feature comparison of discriminative and common blobs within SuperResNET classes. Results from applying the PC3/PC3‐CAVIN1 trained model to the Cav1‐cavin‐1 dataset (30,412 blobs), displayed as a 3×5 grid. First two panels show the proportion of discriminative and common labels within each SRN class (S1A: 0% discriminative; S1B: 26%; S2: 55%; caveolae: 86%). Remaining panels show feature distributions for all samples (blue), as well as discriminative (red) versus common (green) blobs within each class. For S1B and S2 scaffolds, discriminative blobs exhibit lower network density, higher distance to centroid statistics, larger spatial ranges, and greater size metrics, with network modularity differences very pronounced in S1B. Common caveolae show larger values across size‐related features than discriminative caveolae. S1A scaffolds excluded due to absence of discriminative blobs. Box plots show median (center line), interquartile range (box), and 1.5× IQR (whiskers). Statistical comparisons were performed using the two‐sided Mann–Whitney U test. Significance levels: 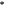 *p* < 0.05, 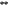 *p* < 0.01, 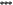 *p* < 0.001. Nonsignificant comparisons (*p* ≥ 0.05) are not marked.

Examination of BOP across SuperResNET classes showed discriminative blobs had higher BOP values than common blobs within each SRN class (Figure 11C). This difference was most pronounced for S1B and S2 scaffolds, while caveolae showed similar BOP for both populations. SuperResNET classification analysis showed 86% of caveolae were identified as discriminative, compared to 55% of S2 scaffolds, 26% of S1B scaffolds, and 0% of S1A scaffolds (Figure 12). Discriminative caveolae were smaller with fewer localizations than common caveolae. Discriminative S1B and S2 scaffolds showed lower network density, higher distance to centroid values, larger spatial ranges, and greater size metrics compared to common structures, with network modularity differences most pronounced in S1B.

These results demonstrate that siMILe, using only weak supervision, identifies structures with enhanced cavin‐1 interaction that include not only the expected caveolae but also S1B and S2 scaffold populations.

### 2.9 Identification of Discriminative Clathrin‐Coated Pits Upon Small Molecule Inhibition

To demonstrate the flexibility and generality of siMILe, we applied siMILe to the previously published SMLM dataset clathrin‐coated pits imaged by labeling the clathrin heavy chain in HeLa cells treated with the clathrin endocytosis inhibitors pitstop 2, dynasore or latrunculin A (LatA) [35]. See summary of data analyzed in Table 1.

**TABLE 1:** Summary of the clathrin endocytosis dataset from Wong et al. [35].

Compared conditions		Number of cells in each replicate (=Total)	Number of training samples	Number of validation samples	Number of testing samples	Total number
DMSO vs Pitstop 2	DMSO	6 + 7 + 8 + 6 + 7 + 6 (=40)	11 363	2418	2176	15 957
	Pitstop 2	7 + 7 + 6 + 6 + 6 (=32)	6529	1526	2647	10 702
DMSO vs LatA	DMSO	6 + 7 + 8 + 6 + 7 + 6 (=40)	11 363	2418	2176	15 957
	LatA	7 + 7 + 7 (=21)	1250	850	1280	3380
DMSO vs Dynasore	DMSO	6 + 7 + 8 + 6 + 7 + 6 (=40)	11 363	2418	2176	15 957
	Dynasore	7 + 7 + 7 (=21)	3529	2474	2945	8948

Clathrin‐mediated endocytosis proceeds through various stages, in which cargo binding initiates the formation of a clathrin‐coated pit that matures, resulting in membrane invagination and dynamin‐dependent scission of the vesicle from the plasma membrane [36]. Small molecule inhibitors of clathrin‐mediated endocytosis include pitstop 2, dynasore, and the actin depolymerizing agent LatA [37, 38]. Pitstop 2 binds directly to the N‐terminal domain of the clathrin heavy chain [39], dynasore is a noncompetitive inhibitor of dynamin, required for clathrin vesicle scission [40, 41], while latrunculin A (LatA) directly binds to actin monomers to inhibit F‐actin polymerization inhibiting clathrin endocytosis [38]. Prior application of SuperResNET analysis to clathrin‐coated pits imaged by labeling the clathrin heavy chain in HeLa cells identified clathrin structures corresponding to clathrin pits and vesicles (class II) [35]. SuperResNET classification feature analysis showed that dynasore and pitstop 2 reduced the size of clathrin pits and vesicles compared to control cells with pitstop 2 more significantly reducing the size of clathrin pits and vesicles compared to dynasore‐treated pits.

**FIGURE 13:**
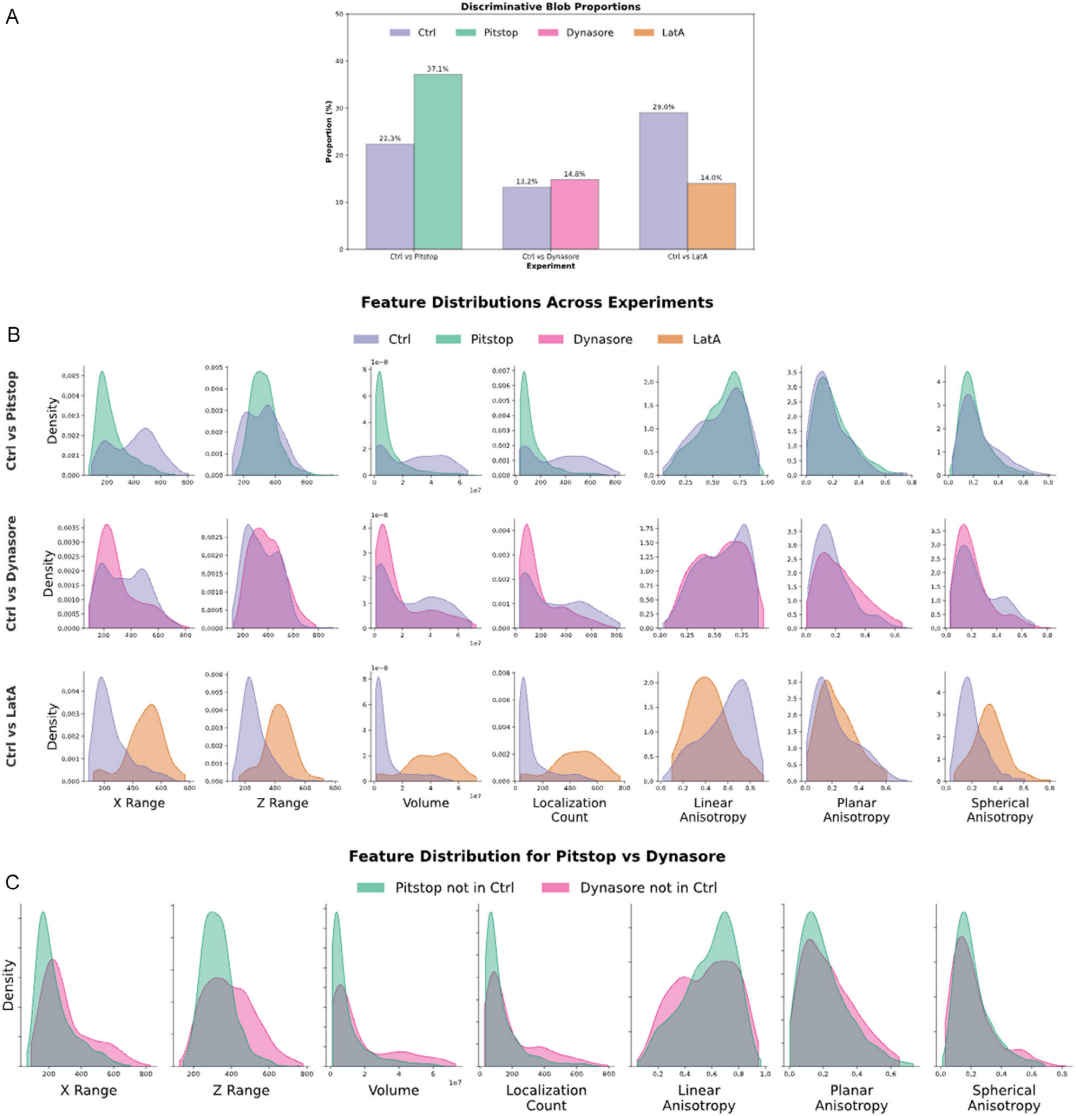
Applying siMILe to clathrin endocytosis datasets to contrast different treatments: Pitstop, Dynasore, and LatA to the control (Ctrl) condition. The SMLM datasets are analyzed with SuperResNET to extract features of clathrin pits (blobs or clusters). (A) The proportions of discriminative blobs when siMILe was used to compare Ctrl to Pitstop; Ctrl to Dynasore, and Ctrl to LatA. (B) The density distribution of the blob features for the same 3 siMILe experiments. (C) Comparing the feature distribution between blobs that are discriminative to Pitstop (vs. Ctrl) with the ones that are discriminative to Dynasore (vs. Ctrl).

Here, we applied siMILe to extract discriminative blobs in pitstop 2, dynasore, and LatA treated cells compared to untreated HeLa cells. siMILe detects a higher percentage of discriminative blobs for pitstop 2 compared to either dynasore or LatA (Figure 13A). This suggests that pitstop 2 causes a more distinct structural shift in clathrin pits, perhaps related to the fact that pitstop 2 binds directly to the clathrin heavy chain [39]. Consistent with SuperResNET feature analysis [35], discriminative blobs identified by siMILe upon pitstop 2 and dynasore inhibition result in blobs with smaller size features compared to control, while LatA blobs are larger with higher spherical anisotropy (Figure 13B). Comparison of discriminative pitstop 2 and dynasore class II blobs shows that dynasore blobs are distinctly larger in their x, y, and z ranges, have a higher localization count, and are less linear and more spherical (Figure 13C). These features suggest that discriminative dynasore blobs are further along the process of clathrin pit maturation than pitstop 2 blobs, as suggested previously [35]. siMILe is therefore able to detect structural differences to clathrin‐coated pits induced by small molecule inhibitors.

## 3 Discussion

### 3.1 SiMILe: an Improved MIL Approach for SMLM

We introduce siMILe, an enhanced MIL approach incorporating MILES with adversarial erasing, to identify, using weak‐supervised learning only, differences in protein oligomer structures in cells using SMLM. Application of siMILe to SuperResNET‐processed 3D SMLM point cloud data effectively identifies caveolae that are selectively found in PC3 cells expressing cavin‐1 [9, 33]. We validate siMILe using simulated and real data and confirm our findings using known interaction of a second protein (cavin‐1) to illustrate that siMILe detects differential structures with biological basis. The use of MIL to label individual structures in 3D point cloud SMLM data has yet to be explored. Our simulation results support the ability of siMILe to improve the classification of more discriminative instances through its very high recall and the significant increase in MILES recall with the addition of adversarial erasing (Figure 5). The combination of the SC with adversarial erasing allows siMILe to maintain very high precision through many iterations that can conservatively label. While MILES can accurately identify discriminative structures when analyzing larger datasets (high precision), it tends to miss many actual discriminative structures (low recall)—a limitation that siMILe overcomes through its combined approach.

Overall, the consistently high F1 score regardless of bag size supports siMILe improvements over MILES. The computational load of siMILe increased significantly with adversarial erasing when maximizing classification performance. We do however, observe, a reduction in this time based on the use of the SC. Finally, across all metrics, the standard deviations of siMILe results are very low, almost always the lowest. This indicates that the contributions cause improved stability in siMILe, alleviating sensitivity to parameters. Computation time is longer as the bag size increases when using adversarial erasing. This shows that only a few discriminative instances are required to perform bag classification. As the bag size gets larger, it takes more iterations to remove all discriminative instances, whereas smaller bag sizes allow more of these instances to be reflected in their bag embedding and during training, leading to correct labeling of them during the instance classification step. It could be claimed that a different solution to this problem would be to use only small bag sizes instead of adversarial erasing. The issue with this approach is that it is only possible when the data is well understood and the witness rate known; otherwise, it cannot be determined which bag size contains few enough discriminative instances. This requirement is reasonable in some applications, but not always possible in discovery.

### 3.2 Application of siMILe to SMLM Datasets

SuperResNET previously selectively identified caveolae in PC3 prostate cancer cells transfected with cavin‐1 [9]. Using the same dataset, we now show that siMILe identifies caveolae as discriminative structures in PC3‐CAVIN1 cells, demonstrating its ability to detect known structures unique to cavin‐1 transfected PC3 cells. siMILe also detected higher order S1B and S2 scaffolds as distinct but not isolated 8S complexes. The 8S complex is a highly stable, SDS‐resistant grouping of 11 Cav1's that exhibits a barrel‐like shape by cryoEM [42, 43]. The 8S complex was identified as S1A scaffolds by SuperResNET and shown to be modules that combine to form higher order scaffolds and caveolae [11]. That no S1A complexes were found to be unique to PC3‐CAVIN1 cells highlights the stable structure of the 8S complex and the robust ability of siMILe to selectively detect discriminatory structures between datasets.

Extension of the approach to a new dataset in which PC3‐CAVIN1 cells are labeled for both Cav1 and cavin‐1 shows that siMILe effectively selects cavin‐1 labeled structures. These include caveolae but also higher order S1B and S2 scaffolds but not 8S complexes (S1A scaffolds). Caveolae show a close association with cavin‐1 with siMILe identifying 80% of SuperResNET defined caveolae as discriminatory. Feature analysis highlights the larger size of common caveolae structures, suggesting that these might be closely associated, overlapping, and unsegmented caveolae that are classed by SuperResNET as caveolae based on size features. Discriminatory S1B and S2 scaffolds were found to more closely associate with cavin‐1 than common S1B and S2 and present larger and more spherical shape features (Figure 12). This suggests that cavin‐1 association with these oligomeric 8S complexes impacts their structure and their organization.

Cavin‐1 is thought to selectively associated with large 70S Cav1 oligomers at the plasma membrane [33, 44]. Our data show that cavin‐1 can associate with higher order 8S oligomers, localized to the plasma membrane by TIRF microscopy, and suggests that progressive association of cavin‐1 with 8S complex oligomers contributes to caveolae formation. siMILe therefore identifies caveolae from cavin‐1 expressing PC3 cells more effectively than SuperResNET and identifies distinct conformations of 8S complex oligomers that are associated with cavin‐1.

In addition, we applied siMILe to a clathrin heavy chain SMLM data set treated with clathrin endocytosis inhibitors [35]. siMILe effectively identified smaller clathrin pits and vesicles induced by treatment with pitstop 2 and dynasore, that prevent clathrin pit maturation and vesicle scission, respectively, and larger ones induced by treatment with the actin depolymerization agent LatA, as previously reported [35, 38, 40].

siMILe is a novel MIL‐based algorithm that incorporates MILES and adversarial erasing to identify discriminatory structures based on SuperResNET SMLM data analysis. siMILe is therefore able to discover novel changes in protein oligomer structure conditional on cell type, genomic, or environmental labels. Uniquely, siMILe is designed to tackle multiple labels without compromising scalability and remains interpretable.

## 4 Conclusion

We introduce siMILe, a novel enhancement on the MIL paradigm, to identify, using weak‐supervised learning only, differences in protein oligomer structures in cells using SMLM. We validate siMILe using simulated and real data, and confirm our findings using known interaction of a second protein (cavin‐1) to illustrate that siMILe detects differential structures with biological basis. siMILe will open the door to novel discovery of changes in protein oligomer structure conditional on cell type, genomic, or environmental labels. Uniquely, siMILe is through its SC better positioned to tackle multiple labels without compromising scalability and remains interpretable when applied to interpretable features.

## 5 Materials and Methods

### 5.1 PC3, PC3‐CAVIN1 Dataset

A previously published SMLM image dataset of human PC3 prostate cancer cells is used [9]. Although PC3 prostate cancer cells express Cav1 (CAV1; UniProtID: Q03135), they do not express cavin‐1 (CAVIN1; UniProtID: Q6NZI2), producing no caveolae. Through stable transfection of cavin‐1 in PC3 cells, caveolae can be induced [33]. The dataset contains the PC3 cells absent of cavin‐1/PTRF (referred to as PC3) and PC3 cells transfected with cavin‐1/PTRF (referred to as PC3‐CAVIN1 cells). The images are acquired over three replicates, each replicate contains 10–11 images; for both PC3 and PC3‐CAVIN1 cells.

### 5.2 Simulated dSTORM Dataset

We generate a simulated dSTORM point cloud dataset for testing siMILe on data with ground truth. The point clouds were generated with the RSMLM software package, used in previous publications to test clustering methods in simulations [45, 46], and the dSTORM simulation parameters were adopted from [46]. We generate the dataset with two classes, A and B, using the setup visualized in Figure 2B. Each class contains clusters representing the structure instances that belong to one of the two classes. The class A contains cluster labels a and c, while the class B contains clusters labeled b and c. The centroid of each cluster is uniformly distributed. The number of localizations is generated using a normal distribution with a mean of 50 and standard deviation of 10. The position of the cluster localizations is also generated with a normal distribution around the centroid, with the c clusters generated with equal standard deviation in x,y,z of 20, whereas the a and b instances differ in their x and z standard deviation, respectively, set at 40. For each class there were 50 cells generated with ∼200 blobs per cell and a witness rate of 10%.

### 5.3 Dual‐Channel Cav1‐Cavin‐1 Dataset

#### 5.3.1 Antibodies and Plasmid

Rabbit anti‐Cav1 (#3267) was purchased from Cell Signaling, and mouse anti‐GFP (A11120) was purchased from Invitrogen. Secondary goat anti‐rabbit Alexa Fluor 647 F(ab)’2 (A‐21 246) was purchased from Invitrogen, and goat anti‐mouse CF 568 F(ab)’2 (20109) was purchased from Biotium. PTRF/cavin‐1‐EGFP plasmid was a generous gift from Dr. Michelle Hill (The University of Queensland Diamantina Institute, Brisbane, Australia) [33].

#### 5.3.2 Cell Culture and Transfection

The PC3 cell (RRID: CVCL_0035) clonal line described in previous study [47] was maintained at 37°C, 5% CO2 in RPMI‐1640 medium (Thermo‐Fisher Scientific Inc.) supplemented with 10% fetal bovine serum (FBS, Thermo‐Fisher Scientific Inc.) and 2 mM L‐glutamine (Thermo‐Fisher Scientific Inc.). The cells were passaged using 0.25% Trypsin‐EDTA (Thermo‐Fisher Scientific Inc.) at approximately 70% confluency and were discarded at the 10th passage. The cells were tested regularly for mycoplasma using a PCR kit (Catalog# G238; Applied Biomaterial, Vancouver, BC, Canada)Joshi, 2008. Plasmid transfection was done 24 h after seeding the cells using Lipofectamine 2000 (Life Technologies, Thermo Fisher Scientific) following manufacturer's protocol.

#### 5.3.3 SMLM Preparation and Imaging

Coverslips (No. 1.5 H) were sonicated for 30 min in 1 M potassium hydroxide followed by 30‐minute sonication in 100% ethanol and then rinsed with Milli‐Q water [12]. The cells were fixed 18 h after transfection using 4% paraformaldehyde (PFA) in phosphate‐buffered saline containing 1 mM MgCl2 and 0.1 mM CaCl2 (PBS‐CM) for 15 min, rinsed thrice with PBS‐CM, permeabilized using 0.2% Triton X‐100 diluted in PBS‐CM, incubated with Image‐iT FX Signal Enhancer (Thermo Fisher Scientific) and blocked using BlockAid Blocking Solution (Thermo Fisher Scientific) [12]. The cells were incubated with primary rabbit anti‐Cav1 and mouse anti‐GFP diluted in saline sodium citrate (SSC) buffer containing 1% BSA, 2% goat serum and 0.05% Triton X‐100 overnight at 4°C and then with secondary F(ab’)2‐goat anti‐rabbit Alexa Fluor 647 and F(ab’)2‐goat anti‐mouse CF568. Cells were washed with SSC buffer containing 0.05% Triton X‐100 and post‐fixed with 4% PFA for 15 min. The cells were then incubated with 0.1 μm TetraSpeck Fluorescent Microspheres (Thermo Fisher Scientific) overnight. Before imaging, samples were mounted in freshly prepared blinking buffer containing 10% glucose (Sigma–Aldrich Inc.), 0.5 mg/ml glucose oxidase (Sigma–Aldrich Inc.), 40 μg/mL catalase (Sigma–Aldrich Inc.), 50 mM Tris, 10 mM NaCl and 50 mM β‐mercaptoethanol (βME; Sigma–Aldrich Inc.) in Milli‐Q water and sealed on glass depression slide. Three replicates of dSTORM images (at least 10 randomly selected images per replicate) were acquired using a Leica SR GSD 3D system with a 160 × objective lens (HC PL APO 160 × /1.43, oil immersion), a 642 nm laser line, a 542 nm laser line and a EMCCD camera (iXon Ultra, Andor). Epi‐illumination was used to bring fluorophores to single molecule blinking, and TIRF‐illumination with 150 nm penetration depth was used for acquisition using Leica Application Suite × using high power mode (region of interest 18×18 μm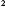). The Alexa Fluor 647 channel was acquired prior to CF568 channel, and each channel was imaged for 4 min with 11 ms exposure per frame. 3D localization was performed using a custom written macro of ImageJ plug‐in ThunderSTORM [48], and lateral drift was corrected using cross correlation. 647 and 568 channels were aligned using SMLMTools.jl [49] using TetraSpeck as a reference.

### 5.4 SuperResNET Merging and Filtering

We used the SuperResNET network analysis tool [9] to preprocess the SMLM point cloud data for all the datasets used. The program iteratively merges blinks within a predefined threshold to correct for multiple‐blinking fluorophores; we used a threshold of 10 nm for the simulated dataset, a threshold of 20 nm for the PC3, PC3‐CAVIN1 dataset, and a threshold of 14 nm for both channels in the Cav1‐cavin‐1 dataset. This is followed by a reduction of background noise not associated with any molecules through a filtering stage by comparing the degree graph of the localizations to that of a random graph with similar statistics. The degree graph was constructed using a proximity distance of 80 nm for both real datasets to determine the neighbors, while 60 nm was used for the simulated dataset. A parameter α is used to determine the filter cutoff. Given $D_{r}$ as the mean degree in the random graph, the localizations that contain a degree less than or equal to the cutoff $\alpha \cdot D_{r}$ are removed. For the PC3‐CAVIN1 dataset, this value was set to α=4. In the Cav1‐cavin‐1 dataset, the Cav1 channel used α=0.5, while the cavin‐1 channel used α=1.95. This was determined by choosing an α that removed 95% from the background of manually segmented cells.

### 5.5 SiMILe Hyperparameters

For the PC3, PC3–CAVIN1 dataset, within each condition and for each experimental replicate we reserved 2 cells for the validation set and 2 cells for the test set, using the remaining cells for training. Hyperparameters (bag size = 500, σ=1000, C=1000, minacc=0.85) were selected by optimizing performance on the validation set and then fixed for evaluation on the held‐out test set.

For the simulated dataset and its ablation study (siMILe, MILES + AE, MILES + SYM‐C, MILES), we employ nested 5‐fold cross‐validation: an inner 5‐fold grid search over the hyperparameter grid in Table 2, and an outer 5‐fold loop to estimate held‐out performance.

**TABLE 2:** Grid of candidate values for inner‐loop tuning on the simulated dataset.

Hyperparameter	Candidate values
Bag size	{5, 25, 50, 75, 100}
σ	{0.1, 0.5, 1, 10, …, $10^{6}$}
C	{0.01, 0.1, 0.5, 1, …, $10^{4}$}
minacc	{0.5, 0.55, …, 0.95}

## Supporting Information

Additional supporting information can be found online in the Supporting Information section.

## Funding

This study was supported by Canadian Institute of Health Research; Digital Research Alliance of Canada; Natural Sciences and Engineering Research Council of Canada.

## Conflicts of Interest

The authors declare no conflicts of interest.

## Supporting information

Supplementary Material

## Data Availability

The data that support the findings of this study are available from the corresponding author upon reasonable request.
