# Supplementary material for: Adversarial Erasing Enhanced Multiple Instance Learning (siMILe): Discriminative Identification of Oligomeric Protein Structures in Single Molecule Localization Microscopy
Source: Adv Intell Syst. 2026 Apr 30;8(7):e202501159. doi: 10.1002/aisy.202501159 (PMC13404161; doi:10.1002/aisy.202501159)
Supplement: Supplementary file 1 — Supplementary Material [file AISY-8-e202501159-s001.pdf]

Supplementary Material

Supervised Learning Baseline

We first examined whether a standard supervised classifier could identify discriminative structures when trained with condition-level labels propagated to all instances. We trained a linear SVM with L1 regularization using the 30 SuperResNET blob features from the simulated dataset, labeling each blob according to its parent condition (A or B). The regularization parameter C was selected via grid search over $\{0.01, 0.1, 1, 10, 100, 1000\}$. To avoid data leakage from correlated blobs within the same simulated image, we performed file-level splitting, holding out entire images rather than individual blobs for testing.

The supervised classifier achieved only 55.2% accuracy in distinguishing conditions, marginally above random chance (50%), reflecting that the majority of blobs (type c) are common to both conditions and provide no discriminative signal. To assess whether the classifier could nonetheless identify discriminative structures, we examined its high-confidence predictions at varying thresholds (Table S1). At a confidence threshold of 0.7, the classifier achieved 90.2% precision but only 45.6% recall in identifying truly discriminative blobs (types a and b). Increasing the threshold to 0.8 improved precision to 100% but reduced recall to just 4.4%.

Table S1: Supervised baseline performance at varying confidence thresholds. Precision and recall are computed for identifying discriminative blobs (types a or b) among high-confidence predictions.

Confidence Threshold	Precision	Recall
0.5	0.097	0.961
0.6	0.346	0.851
0.7	0.902	0.456
0.8	1.000	0.044

These results reveal fundamental limitations of the supervised approach for structure discovery. Even at the threshold achieving reasonable precision (0.7), recall remains below 50%, meaning the majority of discriminative structures go unidentified. This is particularly problematic when multiple types of discriminative structures exist with varying prominence in feature space; the classifier may preferentially identify the most distinctive structures while missing subtler but biologically important ones.

For biological applications where the goal is to identify all condition-specific structures, including those that may be less prominent or previously unknown, comprehensive recall is essential. This motivates the use of multiple instance learning, which explicitly models the relationship between bag-level labels and instance-level predictions. Moreover, siMILe's adversarial erasing mechanism specifically addresses the challenge of comprehensive discovery by iteratively identifying discriminative structures beyond the most prominent ones.

Guide to Users

siMILe's applicability to other datasets depends on two separable components: (1) feature extraction, which is domain-specific, and (2) the core MIL algorithm, which is domain-agnostic. The base algorithm (MILES), that siMILe extends, has been successfully applied across domains including drug discovery, computer vision, and histopathology. More importantly, our key innovations—adversarial erasing and symmetric classification—address fundamental MIL challenges (unknown witness rates and multi-condition discovery) that exist across domains, not just for Caveolae or even SMLM.

siMILe can analyze any dataset where: (1) there are structures/objects from two or more experimental conditions; (2) one can extract numerical features describing these structures; and (3) the goal is to identify condition-specific differences.

Practically, using siMILe requires having access to cells/images from 2 or more different biological conditions or experimental setups (e.g. wild-type/control, genetic mutation, over/under-expression of a protein, pathological condition) that need to be contrasted. From each of these cells/images, the user of siMILe should collect different features that describe the cellular structures, e.g. area/volumes, count, sphericity, border irregularity, etc. This feature extraction can be done with ImageJ, deep learning embeddings, or any domain-appropriate method. In this work, the interactive GUI-based SuperResNET software was the SMLM-specific feature extractor we adopted, which has been used to analyze various cellular structures in previous works [1, 2, 3].

Once features are collected from both conditions, they are collected into CSV files, where each row contains the features as well as an entry for the class (condition) of the cell, e.g. condition 1 vs. condition 2. siMILe expects the setting of the following parameters:

- **σ (sigma)** — Controls similarity bandwidth in the MILES embedding. Larger σ values create broader similarity, making the algorithm more conservative in labeling instances as discriminative. If over-iteration occurs, consider increasing σ substantially.

- **min_acc threshold** — Determines the stopping criterion for adversarial erasing iterations. The default threshold may be too permissive for some datasets. Increasing this value will terminate iterations earlier, preventing over-labeling of instances.

- **C parameter** — Controls regularization strength in the L1-SVM. High C values can cause overfitting to noise, leading to spurious discriminative patterns. Reducing C increases regularization and creates smoother decision boundaries.

- **bag_size** — Determines the number of instances per bag during MILES training. Smaller bags can amplify noise effects. Increasing bag_size provides better statistical averaging and reduces sensitivity to outliers.

siMILe takes the CSV file and the parameters settings and runs its optimization to identify which sub-cellular structures, which rows of the CSV, are discriminant to each class or indiscriminatory.

Detailed usage instructions are provided on the siMILe GitHub repository: `https://github.com/NanoscopyAI/siMILe`.

Algorithm Pseudocode

Algorithm S1 **siMILe Pipeline** for identifying discriminative structures in SMLM data. Point clouds are segmented into blobs using SuperResNET, features are extracted for each blob, and the siMILe algorithm classifies each blob as discriminative to condition 0, discriminative to condition 1, or common to both.

Input:
  $P = \{P_i \mid 1 \leq i \leq n\}$ ▷ Set of n point clouds
  $Y = \{Y_i \in \{0, 1\} \mid 1 \leq i \leq n\}$ ▷ Condition labels
Output:
  $O = \{(Q_i, \hat{Y}_i) \mid 1 \leq i \leq n\}$ ▷ Segmented structures with instance labels

$(Q, F) \leftarrow \{\text{SuperResNET}(P_i) \mid P_i \in P\}$ ▷ Denoise, merge, segment, extract features
$\hat{Y} \leftarrow \text{siMILe}(F, Y)$ ▷ Algorithm S2
return $O \leftarrow \{(Q_i, \hat{Y}_i) \mid 1 \leq i \leq n\}$

Algorithm S2 **siMILe: Instance Classification with Adversarial Erasing.** The algorithm iteratively trains a bag classifier, computes instance classification scores, and uses k-means clustering to identify discriminative instances from both conditions. Identified instances are removed (adversarial erasing) and the process repeats until bag classification accuracy falls below the threshold.

Input:
    $F = \{F_i \mid 1 \leq i \leq n\}$ where $F_i = \{f_{ij} \in \mathbb{R}^{30}\}$ ▷ Features per cell
    $Y = \{Y_i \in \{0, 1\} \mid 1 \leq i \leq n\}$ ▷ Condition labels
    $\sigma, C, \text{bag\_size}, \text{min\_acc}$ ▷ Hyperparameters
Output:
    $\hat{Y} = \{\hat{Y}_i\}$ where $\hat{Y}_i = \{\hat{y}_{ij} \in \{-1, 0, 1\}\}$ ▷ Instance labels

$\hat{Y} \leftarrow \{\}$ ▷ Initialize accumulated predictions
$\text{acc} \leftarrow 1.0$
while $\text{acc} > \text{min\_acc}$ **do**
    $w^*, b^* \leftarrow \text{TrainMILES}(F, Y, \sigma, C, \text{bag\_size})$ ▷ Alg. S3
    Construct bags B and embeddings E as in Algorithm S3
    ▷ Compute instance classification scores
    for each bag $B_i \in B$ **do**
        $I \leftarrow \{k \mid |w_k^*| > 0\}$ ▷ Non-zero weight indices
        for each instance $b_{ij} \in B_i$ **do**
            $I_j \leftarrow \{k \in I \mid j =_{j'} \|b_{ij'} - c_k\|\}$ ▷ Concepts closest to b_{ij}
            $m_k \leftarrow |\{j' \mid k \in I_{j'}\}|$ ▷ Instances sharing concept c_k
            $g(b_{ij}) \leftarrow \sum_{k \in I_j} \frac{w_k^* \cdot \exp(-\|b_{ij} - c_k\|^2/\sigma^2)}{m_k}$
        end for
    end for
    ▷ Symmetric classification via k-means
    $t_0, t_{\text{null}}, t_1 \leftarrow \text{kmeans}_3(\{g(b_{ij}) \mid \forall i, j\})$ ▷ 3 cluster centers
    Sort centers: $t_0 < t_{\text{null}} < t_1$
    for each instance b_{ij} **do**
        $c \leftarrow_{t \in \{t_0, t_{\text{null}}, t_1\}} |g(b_{ij}) - t|$
        $\hat{y}_{ij} \leftarrow \begin{cases} 0 & \text{if } c = t_0 \\ 1 & \text{if } c = t_1 \\ -1 & \text{otherwise} \end{cases}$
    end for
    $\hat{Y} \leftarrow \hat{Y} \cup \{\hat{y}_{ij} \mid \hat{y}_{ij} \neq -1\}$ ▷ Accumulate discriminative
    Remove instances with $\hat{y}_{ij} \neq -1$ from F ▷ Adversarial erasing
    $\text{acc} \leftarrow \text{BagAccuracy}(w^*, b^*, E, Y_B)$
end while
return $\hat{Y}$

Algorithm S3 TrainMILES: Bag Classifier Training. Instances are pooled by condition and all serve as concepts. Bags are constructed by randomly grouping instances within each condition. Each bag is embedded by computing its maximum similarity to each concept, and an L1-regularized linear SVM is trained on these embeddings.

Input:
  $F = \{F_i\}$ ▷ Feature sets per cell
  $Y = \{Y_i \in \{0, 1\}\}$ ▷ Cell labels
  σ, C, bag_size ▷ Hyperparameters
Output:
  w^*, b^* ▷ SVM weights and bias

$f_0 \leftarrow \bigcup\{f_{ij} \mid f_{ij} \in F_i \wedge Y_i = 0\}$ ▷ All instances from condition 0
$f_1 \leftarrow \bigcup\{f_{ij} \mid f_{ij} \in F_i \wedge Y_i = 1\}$ ▷ All instances from condition 1
$\mathcal{C} \leftarrow f_0 \cup f_1$ ▷ All instances serve as concepts
$B_0 \leftarrow$ CreateBags(shuffle(f_0), bag_size) ▷ Random bags from condition 0
$B_1 \leftarrow$ CreateBags(shuffle(f_1), bag_size) ▷ Random bags from condition 1
$B \leftarrow B_0 \cup B_1$; $Y_B \leftarrow$ corresponding bag labels
$E \leftarrow \{\}$ ▷ Bag embeddings
for each bag $B_i \in B$ **do**
  for each concept $c_k \in \mathcal{C}$ **do**
    $e_{ik} \leftarrow \max_{b_{ij} \in B_i} \exp\left(\frac{-\|b_{ij} - c_k\|^2}{\sigma^2}\right)$
  end for
  $E_i \leftarrow [e_{i1}, e_{i2}, \ldots, e_{i|\mathcal{C}|}]$ ▷ Bag embedding vector
end for
$w^*, b^* \leftarrow$ L1-SVM(E, Y_B, C) ▷ Train linear SVM with L1 regularization
return w^*, b^*

Table S2: SuperResNET Blob Features

Feature Name	Description	Formula
Basic Statistics		
Number of localizations	Total number of localizations in blob	$\lvert b_i \rvert = N$
Average distance to centroid	Mean distance of localizations to blob center	$\bar{R_{c_i}} = \frac{\sum_{j=1}^{N} d_j}{N}$
Minimum distance to centroid	Smallest distance to blob center	$MinR_{c_i} = \min(R_c)$
Maximum distance to centroid	Largest distance to blob center	$MaxR_{c_i} = \max(R_c)$
Median distance to centroid	Middle value of distances to center	$MedR_{c_i} = \text{median}(R_c)$
Std dev of distance to centroid	Spread of distances from center	$StdR_{c_i} = \sqrt{\frac{\sum_{j=1}^{N}(d_j - \bar{R}_{ci})^2}{N}}$
Shape and Anisotropy Features		
Fractional anisotropy (FA)	Degree of anisotropy of blob diffusion	$FA_i = \sqrt{\frac{3((\lambda_1-\bar{\lambda})^2+(\lambda_2-\bar{\lambda})^2+(\lambda_3-\bar{\lambda})^2)}{2(\lambda_1^2+\lambda_2^2+\lambda_3^2)}}$
Linear anisotropy (CL)	Elongation along one direction	$CL_i = \frac{\lambda_1-\lambda_2}{\lambda_1}$
Planar anisotropy (CP)	Restriction to plane	$CP_i = \frac{\lambda_2-\lambda_3}{\lambda_1}$
Spherical anisotropy (CS)	Isotropic diffusion measure	$CS_i = \frac{\lambda_3}{\lambda_1}$
Volume	3D convex hull volume	Delaunay triangulation
Network Degree Features		
Average degree	Mean node connectivity	$\bar{deg_i} = \frac{\sum_{j=1}^{N} deg_j}{N}$
Maximum degree	Highest node connectivity	$maxDeg_i = \max(Deg)$
Minimum degree	Lowest node connectivity	$minDeg_i = \min(Deg)$
Network Path and Distance Features		
Characteristic path	Average shortest path length	$charPath_i = \frac{1}{N}\sum_{u\in V_i} L_u$
Average eccentricity	Mean maximum distance between nodes	$e\bar{c}c_i = \frac{1}{N}\sum_{u\in V_i} ecc_u$
Network radius	Minimum network eccentricity	$radius_i = \min(Ecc)$
Network diameter	Maximum network eccentricity	$diameter_i = \max(Ecc)$
Clustering Features		
Average clustering coefficient	Mean fraction of triangles around nodes	$\bar{cc}_i = \frac{1}{N}\sum_{u\in V_i} cc_u$
Maximum clustering coefficient	Highest clustering value	$maxCC_i = \max(CC)$
Minimum clustering coefficient	Lowest clustering value	$minCC_i = \min(CC)$
Network Structure Features		
Network density	Fraction of actual to potential connections	$netDen_i = \frac{E_i}{PC_i}$
Network transitivity	Ratio of triangles to triplets	$trans_i = \frac{\sum_{u\in V_i} 2tri_u}{\sum_{u\in V_i} deg_u(deg_u-1)}$
Network modularity	Strength of network subdivision	$mod_i = \sum_{u\in M}[e_{uu} - (\sum_{v\in M} e_{uv})^2]$
Optimized modularity	Optimal modular structure	$\arg\max_M\{\lvert E_{intra}\rvert - \lvert E_{inter}\rvert\}$
Spatial Range Features		
X range	Spread along x-dimension	$xrange_i = \max(x_{coords}) - \min(x_{coords})$
Y range	Spread along y-dimension	$yrange_i = \max(y_{coords}) - \min(y_{coords})$
Z range	Spread along z-dimension	$zrange_i = \max(z_{coords}) - \min(z_{coords})$
Other Features		
Hollowness	Relative spread from centroid	$hollowness_i = \frac{mean(R_c)}{std(R_c)}$
Blob area	2D (x,y) area of blob	2D area calculation

Notation: B is the set of all segmented blobs. $b_i \in B$ denotes blob i with localizations $\{p_1, ..., p_N\}$. $d_j = \lvert p_j - c_i \rvert$ is the distance from localization p_j to centroid c_i. $R_c = [d_1, d_2, ..., d_N]$ is the vector of all distances to centroid for blob b_i. $G_i = (V_i, E_i)$ is the proximity graph with threshold PT. $\lambda_1, \lambda_2, \lambda_3$ are PCA eigenvalues with mean $\bar{\lambda}$. Deg, Ecc, CC are vectors containing degree, eccentricity, and clustering coefficient values for all nodes. L_u is the average distance from node u to all other nodes. tri_u is the number of triangles around node u. M denotes network modules with e_{uv} representing edge portions between modules. $PC_i = N(N-1)/2$ is the number of potential connections in the undirected network of blob b_i.

References

[1] Y L* Li, I M* Khater, C Hallgrimson, B Cardoen, T H Wong, G Hamarneh, and I R Nabi. Superresnet single molecule localization microscopy model-free network analysis achieves molecular resolution of nup96. *Advanced Intelligent Systems*, In press, 2024.

[2] Kailasam Mani, Nicolas Tardif, Olivier Rossier, Ismail M. Khater, Xuesi Zhou, Filipe Nunes Vicent, Radhakrishnan Av, Celine Gracia, Pamela Gonzalez Troncoso, Isabel Brito, Richard Ruez, Melissa Dewulf, Ghassan Hamarneh, Ivan Robert Nabi, Pierre Sens, Irina S Moreira, Gregory Giannone, Cedric M Blouin, and Christophe Lamaze. Remote control of cell signaling through caveolae mechanics. Technical Report biorxiv:2024.03.12.584716, Simon Fraser Universiry, 7 2025.

[3] Timothy H. Wong, Ismail M. Khater, Christian Hallgrimson, Y. Lydia Li, Ghassan Hamarneh, and Ivan Robert Nabi. Superresnet – single-molecule network analysis detects changes to clathrin structure induced by small-molecule inhibitors (wong and khater: Joint first authors; hamarneh and nabi: Joint senior authors). *Journal of Cell Science (JCS)*, 138(4):1–11, 2025.
